# Twelve-hour rhythms in transcript expression within the human dorsolateral prefrontal cortex are altered in schizophrenia

**DOI:** 10.1371/journal.pbio.3001688

**Published:** 2023-01-24

**Authors:** Madeline R. Scott, Wei Zong, Kyle D. Ketchesin, Marianne L. Seney, George C. Tseng, Bokai Zhu, Colleen A. McClung

**Affiliations:** 1 Translational Neuroscience Program, Department of Psychiatry, Center for Neuroscience, University of Pittsburgh, Pittsburgh, Pennsylvania, United States of America; 2 Department of Bioinformatics, University of Pittsburgh, Pittsburgh, Pennsylvania, United States of America; 3 Aging Institute of UPMC, University of Pittsburgh School of Medicine, Pittsburgh, Pennsylvania, United States of America; Weizmann Institute of Science, ISRAEL

## Abstract

Twelve-hour (12 h) ultradian rhythms are a well-known phenomenon in coastal marine organisms. While 12 h cycles are observed in human behavior and physiology, no study has measured 12 h rhythms in the human brain. Here, we identify 12 h rhythms in transcripts that either peak at sleep/wake transitions (approximately 9 AM/PM) or static times (approximately 3 PM/AM) in the dorsolateral prefrontal cortex, a region involved in cognition. Subjects with schizophrenia (SZ) lose 12 h rhythms in genes associated with the unfolded protein response and neuronal structural maintenance. Moreover, genes involved in mitochondrial function and protein translation, which normally peak at sleep/wake transitions, peak instead at static times in SZ, suggesting suboptimal timing of these essential processes.

## Introduction

Twelve-hour (12 h) ultradian rhythms have long been observed in coastal marine animals, whose behavior aligns with ocean tides [1]. Recent studies have confirmed 12 h transcriptional rhythms in other organisms including *C*. *elegans*, mice, and olive baboons [1]. Various aspects of human behavior (sleep patterns, cognitive performance) and physiology (body temperature, blood pressure, migraine onset, circulating hormone levels) also exhibit 12 h rhythms [1]. In cases like blood pressure and body temperature, these 12 h rhythms are secondary to a dominant 24 h rhythm, suggesting the presence of multiple superimposed rhythms. However, as 12 h rhythms in transcript expression have not been identified in human tissue, it is unknown whether these processes are related to and/or regulated by molecular ultradian rhythms. Therefore, characterization of the human brain ultradian transcriptome will expand our understanding of transcript expression rhythms in the brain and their contribution to dysfunction in subjects with abnormalities in brain function.

Schizophrenia (SZ) is a chronic neuropsychiatric illness that affects over 20 million people worldwide and is a leading cause of disability [2]. Many SZ patients experience disturbances in the rhythmicity of sleep/wake cycles, peripheral gene expression, and daily hormones [3,4]. Molecular rhythm patterns, however, have only just begun to be directly measured in the human brain. To explore these rhythms in human postmortem brain tissue, our lab and others have utilized a “time of death” (TOD) analysis, in which gene expression data are organized across a 24 h clock based on the time of day of the subject’s death, to identify significant changes in gene expression rhythm patterns associated with specific brain regions [5], age [6], and psychiatric illnesses [7–9]. In SZ subjects, rhythmic analysis of RNA sequencing (RNA-seq) data collected by the CommonMind Consortium [10] from the dorsolateral prefrontal cortex (DLPFC) identified a loss of diurnal rhythmicity in a number of transcripts. Notably, SZ subjects exhibited 24 h rhythmicity in a set of transcripts that were not rhythmic in subjects with no psychiatric diagnosis (NP) [8]. Genes with enhanced 24 h rhythmicity in the SZ cohort were associated with mitochondria dysfunction and GABA-ergic signaling [8], consistent with previous work that finds differential expression of these pathways in subjects with SZ [11,12]. These studies demonstrate that circadian rhythms in gene expression can be reliably measured in human brain tissue and are severely disrupted in the DLPFC of SZ subjects. However, no study to date has attempted to measure 12 h rhythms in transcript expression in human brain or determine if there are changes to these ultradian rhythms in subjects with SZ.

In the current study, we use DLPFC data previously analyzed for circadian rhythms, allowing us to compare both 12 and 24 h rhythms within the same subjects. Multiple convergent analyses identify transcripts that have measurable 12 h rhythms in human DLPFC, with distinct abnormalities in the identity and timing of these transcripts in SZ.

## Results

### Multiple rhythmicity analyses identify 12 h rhythms in human DLPFC

**Fig 1:**
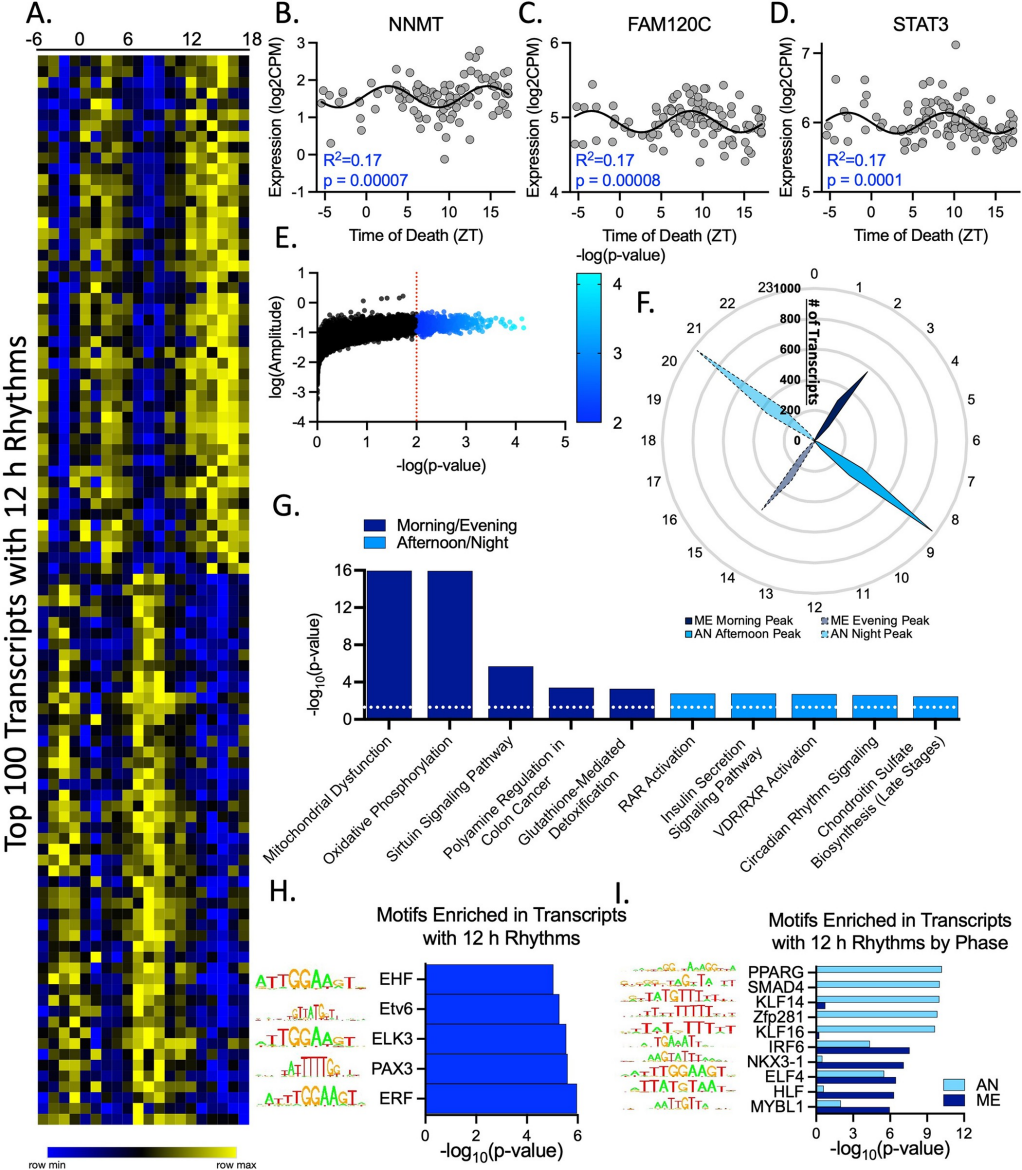
12 h rhythms in the human DLPFC. (**A**-**E**) 12 h rhythms identified by a sinusoidal NLR. (**A**) Heatmap of top 100 transcripts with 12 h rhythms, ordered by phase. (**B**-**D**) Gene expression over TOD scatterplots for the top 3 transcripts with 12 h rhythms showing the sinusoidal curve, goodness of fit (R^2^), and *p*-value. Expression values for these graphs can be found in S6 File. (**E**) Plot comparing NLR-derived amplitude and *p*-values for all transcripts. Transcripts with significant 12 h rhythms in expression are denoted in blue (*p* < 0.01). (**F**) Radar plot showing peak expression times (ZT) of transcripts with 12 h rhythms (*p* < 0.01). Outer scale in ZT (h), radial scale represents number of transcripts, solid lines indicate first peak, and dashed lines indicated second peak. ME peak times indicated in dark blue, AN indicated in light blue. (G) IPA of transcripts with 12 h rhythms separated by peak time. (**H**-**I**) Motif enrichment analysis of transcripts with 12 h rhythms (**H**) all together and (**I**) separated by phase. Results of the NLR, IPA, and motif analyses can be found in S1, S7, and S8 Files, respectively. AN, afternoon/night; DLPFC, dorsolateral prefrontal cortex; IPA, Ingenuity Pathway Analysis; ME, morning/evening; NLR, nonlinear regression; TOD, time of death; ZT, Zeitgeiber time.

We used a modified version of the nonlinear regression (NLR) TOD analysis used previously to determine circadian rhythms [5–9], in which a sinusoidal curve with a 12 h period is fit to gene expression across TOD, to measure 12 h rhythms in the human DLPFC of 104 subjects with known TOD (Figs 1A–1D, S1A and S1B and S1 Table). Out of the 13,914 detected transcripts, 819 (approximately 6%) have significant 12 h rhythms at a threshold of *p* < 0.01, which we have previously used to delineate genes with 24 h rhythms in this cohort [8] (Fig 1E and S2 Table and S1 File). In a bootstrapping analysis of this approach, all genes identified as rhythmic in the original analysis were identified in >50% of the analyses (S2 Fig and S2 File). As a confirmation of the NLR approach, we also employed a Lomb–Scargle analysis, which finds the best fitting sinusoidal curve for each transcript in a manner that is unbiased in terms of period (S3A Fig and S3 File) [13], and an eigenvalue/pencil analysis, which treats temporal gene expression as a composite rhythm and identifies four superimposed rhythmic components (RCs) that, when combined, best explain the temporal expression of each transcript (S3C–S3E and S4 Figs and S4 and S5 Files). In both the Lomb–Scargle and eigenvalue/pencil analyses, we found enrichment of transcripts with 12 h periods (S3A and S3F Fig). Approximately 83% of transcripts identified as having 12 h rhythms in the NLR (*p* < 0.01) had a 12 h period in the Lomb–Scargle analysis (S3B Fig), and 68% had a 12 h RC in the eigenvalue/pencil analysis. Additionally, Ingenuity Pathway Analysis (IPA) found enrichment in mitochondria-related pathways (Oxidative Phosphorylation, Mitochondria Dysfunction, Sirtuin Signaling) for both NLR identified 12 h rhythms and eigenvalue/pencil identified 12 h RCs (S3G Fig and S7 File). The combination of these approaches gave us confidence that we had identified 12 h rhythms in the DLPFC.

### Distinct timing patterns separate 12 h rhythms into two populations

The timing of 12 h rhythms revealed two distinct populations of transcripts, one which peaked in expression in the morning/evening (Zeitgeiber time (ZT) 2 to 3 and 14 to 15; approximately 9 AM/PM) and the other that peaked during the afternoon/night (ZT 8 to 9 and 20 to 21; approximately 3 AM/PM) (Fig 1F). Transcripts associated with mitochondria and the proteasome (Polyamine Regulation in Colon Cancer) were enriched in the morning/evening population, while those associated with the cytoskeleton and calcium signaling (RAR Activation, Insulin Secretion Pathway, VDR/RXR Activation) peaked in the afternoon/night (Fig 1G and S7 File).

**Fig 2:**
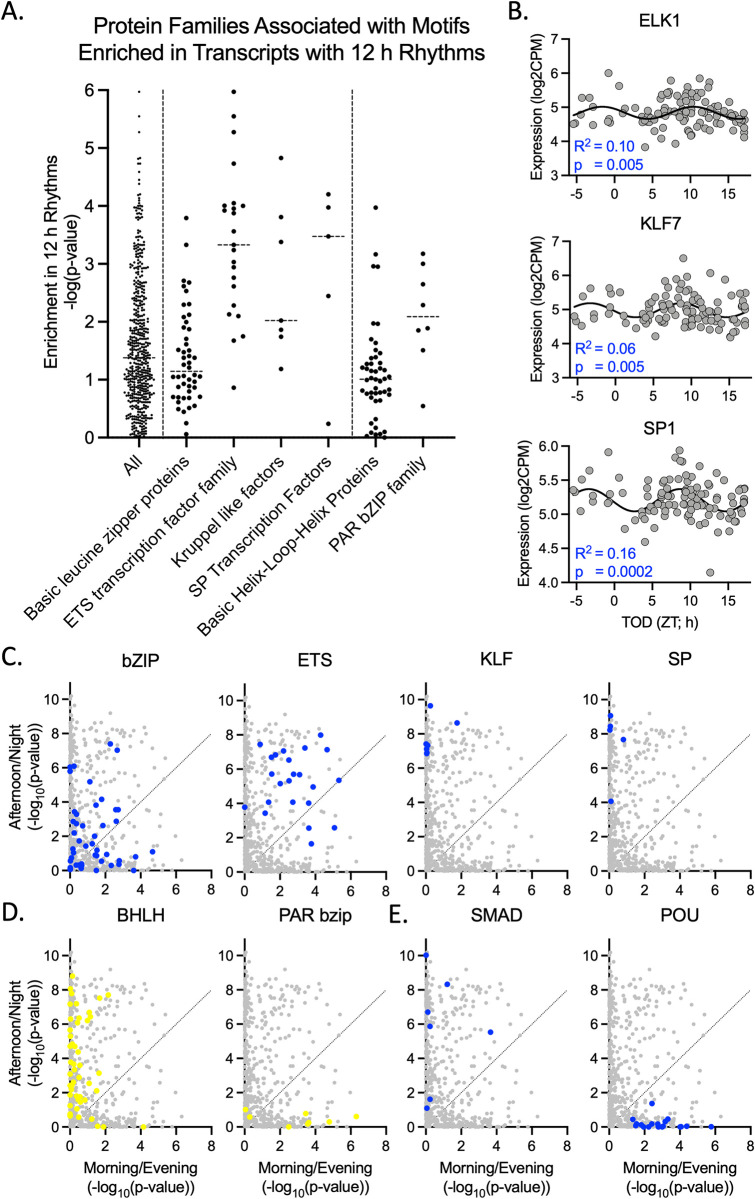
Protein families associated with motifs enriched in transcripts with 12 h rhythms. (**A**) Motif enrichment analysis of transcripts with 12 h rhythms in the human DLPFC. Dashed lines separate three groups: (1) All motifs tested; (2) Protein families previously implicated in regulating 12 h rhythms; (3) Protein families previously implicated in regulating 24 h rhythms. (**B**) Examples of transcription factors from ETS domain (Elk1), Kruppel-like factor (KLF7), and SP transcription factor (SP1) families that have 12 h rhythms in human DLPFC. Expression values for these graphs can be found in S6 File. (**C**-**E**) Comparison of motif enrichment analysis of transcripts with 12 h rhythms that peak in the morning/evening and those that peak in the afternoon/night. (**C**) Protein families previously implicated in regulating 12 h rhythms. Individual motifs are marked in blue for each family. (**D**) Protein families previously implicated in regulating 24 h rhythms. Individual motifs are marked in yellow for each family. (**E**) Other top protein families associated with motif enrichment. Motif analysis results used to create this figure can be found in S8 File. BHLH, Basic Helix–Loop–Helix; bZIP, basic leucine zipper; DLPFC, dorsolateral prefrontal cortex; KLF, Kruppel-like factor; TOD, time of death; ZT, Zeitgeiber time.

We next determined potential sites of regulation and predicted upstream regulators for 12 h rhythms using a motif enrichment analysis [14] (Fig 1H and 1I and S8 File). Motifs enriched in transcripts with 12 h rhythms are bound by transcription factors from the ETS domain family, Kruppel-like factors (KLFs), and the SP domain family (Figs 1H, 2A, and 2B). Motifs associated with the ETS domain family remained enriched when we separately analyzed transcripts that peak in the morning/evening and afternoon/night, but both the KLF and SP domain families were associated only with motifs enriched in the afternoon/night (Figs 1I and 2C). Motifs associated with the circadian-related Basic Helix–Loop–Helix (BHLH) domain family were not enriched in the analysis of transcripts with 12 h rhythms but were strongly enriched when analyzing the afternoon/night group separately (Fig 2D). Alternatively, motifs associated with PAR domain containing basic leucine zipper (bZIP) proteins, which have also been implicated in regulating circadian rhythms [15], were enriched both in the overall analysis (Figs 1H and 2A) and in the morning/evening group (Fig 2D). Other families associated with motifs enriched in the morning/evening include the homeobox POU and PRD classes, while motifs associated with the SMAD family and peroxisome proliferator-activated receptors (PPARs) were enriched in the afternoon/night group (Figs 1I and 2E).

### Subjects with schizophrenia have fewer transcripts with 12 h rhythms

**Fig 3:**
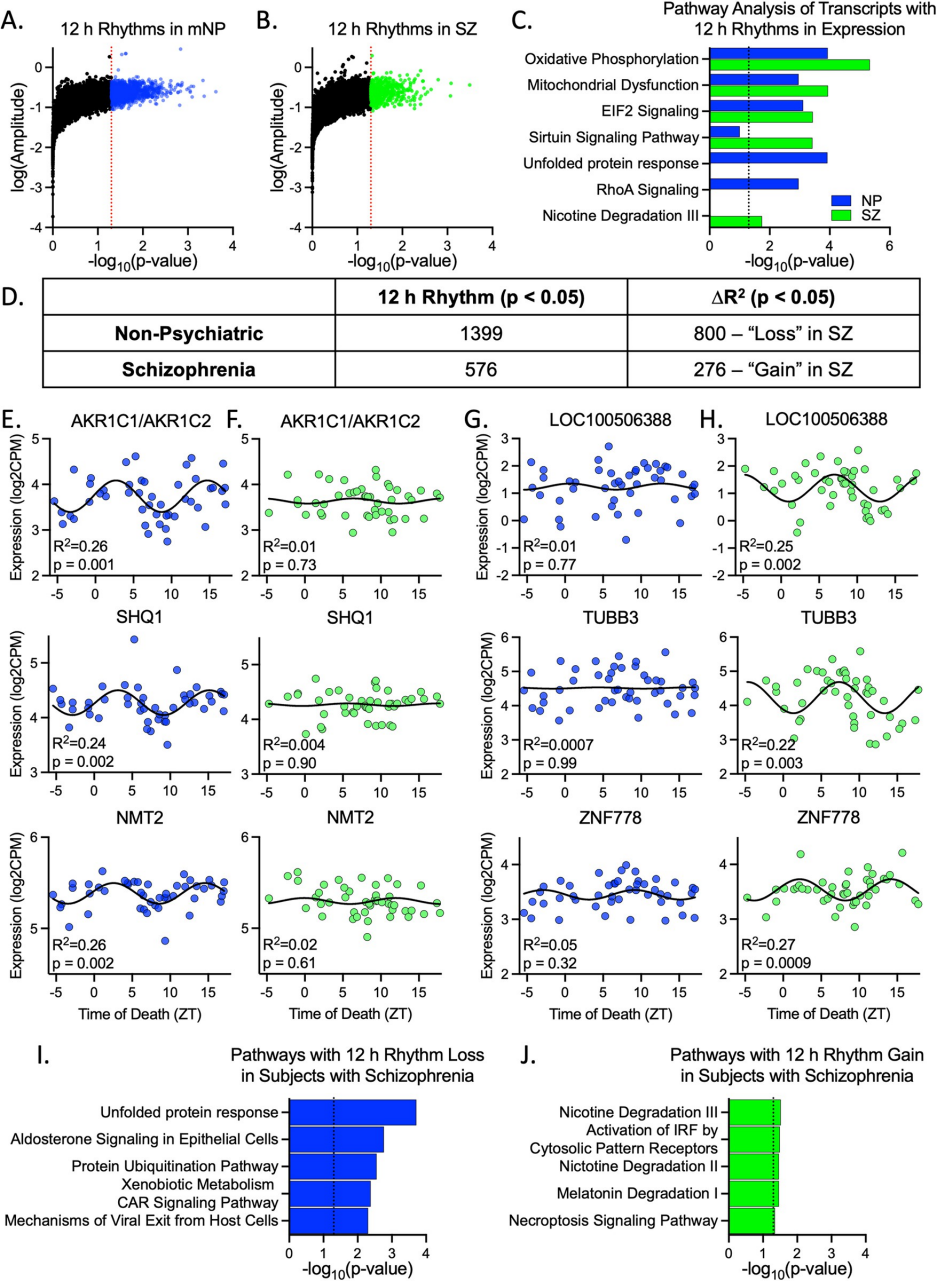
12 h rhythm reprogramming in SZ. NLR analysis identified 12 h rhythms in the (**A**) mNP and (**B**) SZ cohorts. (**C**) IPA of transcripts with significant 12 h rhythms. (**D**) Number of transcripts identified as having a significant 12 h rhythm or a significant difference in goodness of fit (R^2^) between the two cohorts (deltaR^2^). (**E**-**H**) Examples of genes that (**E**-**F**) lose or (**G**-**H**) gain 12 h rhythms in SZ. Expression values for these graphs can be found in S6 File. Gene expression over time is shown for both the (**E**, **G**) mNP and (**F**, **H**) SZ cohorts. (**I**-**J**) IPA of transcripts that significantly (**I**) lose or (**J**) gain rhythmicity in SZ. Results of the NLR and IPA can be found in S1 and S7 Files, respectively. IPA, Ingenuity Pathway Analysis; mNP, match NP; NP, no psychiatric diagnosis; NLR, nonlinear regression; SZ, schizophrenia; ZT, Zeitgeiber time.

We next determined if 12 h rhythms were different in subjects with SZ, a psychiatric illness in which the DLPFC plays a central role [16]. Again, we used existing RNA-seq data produced from the CommonMind Consortium [10] (S1 Table and S1E and S1F Fig). We performed a sinusoidal NLR TOD analysis on a cohort of 46 SZ subjects and a group of 46 NP subjects taken from the full NP (fNP) cohort that best match the SZ cohort for TOD, sex, age, race, pH, and PMI (match NP (mNP); S1 Table and S1C and S1D Fig). Due to sample size limitations, we used a less stringent (*p* < 0.05) statistical cutoff, consistent with our previous circadian analysis of these cohorts [8]. When we employed this cutoff, 1,399 (10%) of transcripts had a 12 h rhythm in expression in the mNP cohort, while only 576 (5%) had a 12 h rhythm in SZ (Fig 3A, 3B, and 3D and S1 File). Bootstrapping analyses confirmed that all genes identified as rhythmic in these original analyses were identified in >50% of the samplings (S2 Fig and S9 and S10 Files). Of these transcripts, only 48 had a significant rhythm in both cohorts. A threshold-free approach, rank-rank hypergeometric overlap (RRHO) [17] (S6 Fig) confirmed that rhythmicity in the mNP cohort was very similar to the fNP cohort. Additionally, we observe some overlap between the mNP and SZ cohorts—but not among the top rhythmic genes. Despite the lack in direct overlap, EIF2 signaling and mitochondria-associated pathways were the top pathways associated with transcripts showing 12 h rhythms for both the mNP and SZ cohorts (Fig 3C and S7 File). Notably, only mNP subjects had 12 h rhythms in transcripts associated with the unfolded protein response (UPR) and RhoA Signaling (Fig 3C and S7 File).

We next performed a loss/gain analysis of 12 h rhythmicity in SZ, as described previously for 24 h rhythms [8], to confirm our finding of fewer 12 h rhythms in SZ and to determine if transcripts experience ultradian reprogramming in SZ. A total of 800 transcripts significantly lost 12 h rhythmicity, and 276 transcripts gained rhythmicity in SZ (Fig 3D-3H and S1 File). Overall, transcripts that lost rhythmicity were associated with the UPR and the Protein Ubiquitination Pathway, while the smaller number of transcripts that gained rhythmicity did not fall into clear pathways (Fig 3I and 3J and S7 File).

### 12 and 24 h rhythms converge on mitochondria-associated pathways in schizophrenia

**Fig 4:**
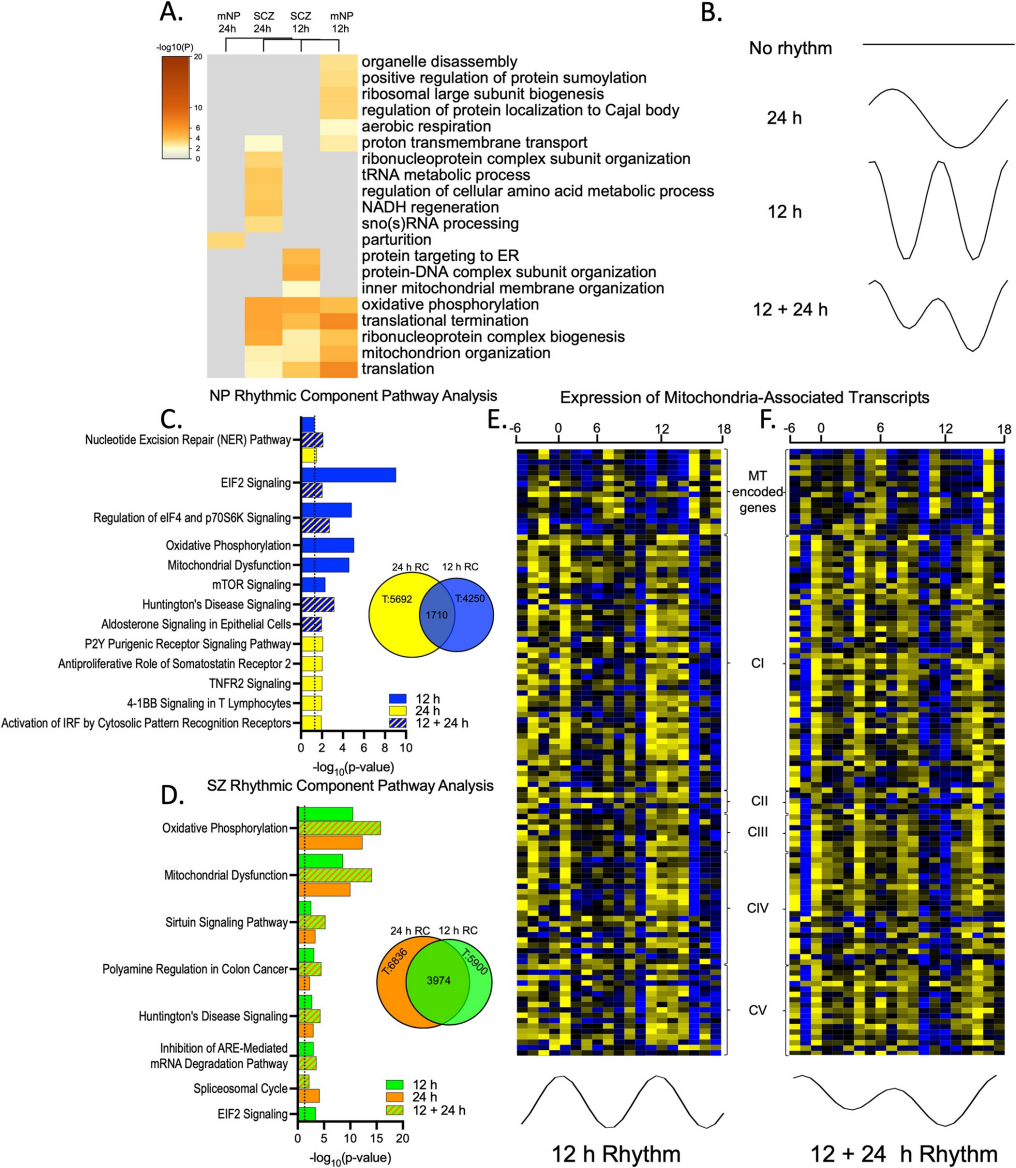
12 and 24 h rhythms converge on mitochondria-associated transcripts. (**A**) Metascape-derived heatmaps of biological processes enriched for 12 and 24 h rhythms in mNP and SZ cohorts. Full Metascape results can be found in S11 File. (**B**) Illustrations of how transcripts could have no rhythms, a 24 h rhythm, a 12 h rhythm, or a combined 12 and 24 h rhythm. Results of eigenvalue/pencil analysis for mNP and SZ subjects can be found in S12 and S13 Files, while a summary of the 12 and 24 h RCs is detailed in S5 File. (**C**-**D**) IPA of transcripts with 12 h and 24 h RCs in (**C**) mNP subjects and (**D**) subjects with SZ. IPA results can be found in S7 File. (**E**-**F**) Gene expression heatmaps of subunits from the MT electron transport chain complexes (CI-CV) across time of day from (**E**) mNP subjects, which show a 12 h rhythm, and (**F**) subjects with SZ, which have a 12 + 24 h rhythm. IPA, Ingenuity Pathway Analysis; mNP, match NP; MT, mitochondria; RC, rhythmic component; SZ, schizophrenia.

Our previous work found a surprising gain of 24 h rhythmicity in mitochondria-related transcripts in subjects with SZ compared to the NP group [8]. Here, we expanded upon those results using Metascape [18] to determine the biological processes implicated in the top transcripts with significant 12 and 24 h rhythms in mNP subjects and subjects with SZ (Figs 4A and S7 and S11 File). While each group had unique aspects of biological process enrichment, mitochondria- and translation-associated biological processes were enriched for both 12 and 24 h rhythms in SZ, but only 12 h rhythms in the mNP cohort (Figs 4A and S7 and S11 File).

We next utilized the eigenvalue/pencil analysis, which allowed us to identify transcripts with gene expression that were best explained by either a single 12 h RC, a single 24 h RC, or a combination of both 12 and 24 h RCs (Fig 4B). Notably, the eigenvalue/pencil analysis identifies RCs—not overall rhythms in expression—and does not incorporate *p*-values [19]. This results in far more transcripts identified as having a 12 h RC by the eigenvalue/pencil analysis than a 12 h rhythm by the NLR analysis. Despite this, a very similar pattern in pathway enrichment emerged between the two analyses. In the mNP cohort, transcripts with 12 and 24 h RCs were found in distinct pathways (Fig 4C and S7 and S12 Files), while both 12 and 24 h RCs were linked to mitochondria-related pathways in SZ (Fig 4D and S7 and S13 Files). Intriguingly, the number of transcripts with both 12 and 24 h RCs increased from 1,710 (30% and 40% of 24 and 12 h RCs, respectively) in the mNP cohort to 3,974 (58% and 67% of 24 and 12 h RCs, respectively) in the SZ cohort (Fig 4C and 4D and S5 File). As an example of what these data show, expression of transcripts in the electron transport chain complexes across time indicated mitochondria-associated genes had 12 h rhythms in the mNP cohort, while in SZ, these transcripts had a combination of 12 and 24 h RCs (Fig 4E and 4F). We propose that this may explain why the NLR analysis identifies this group of transcripts as either a 12 h or 24 h rhythm, or both, in SZ subjects.

### Altered timing of transcripts with 12 h rhythms in schizophrenia

**Fig 5:**
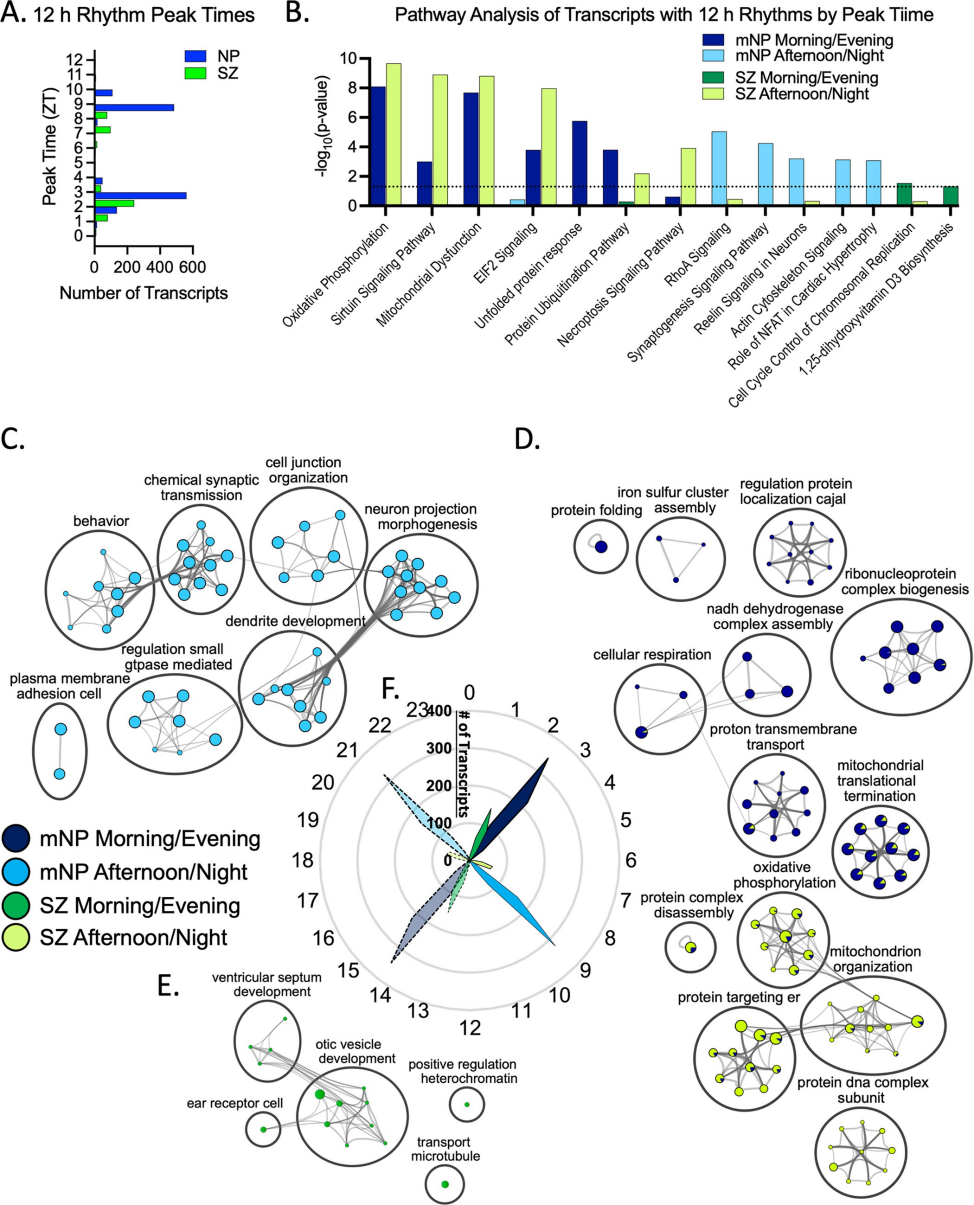
Altered timing of 12 h rhythms in schizophrenia. (**A**) Histogram of 12 h rhythm peak times in mNP and SZ. (**B**) IPA of transcripts with 12 h rhythms split by peak time. (**C**-**E**) Cytoscape visualization [20] of a Metascape analysis demonstrating the biological processes enriched for 12 h rhythms that peak during the morning/evening or afternoon/night. (**F**) Radar plot showing number of transcripts that peak at each time of day. Outer scale is ZT (h) time, radial scale is number of transcripts, solid lines indicate first peak, and dashed lines indicate second peak. Results of the NLR, IPA, and Metascape analyses can be found in S1, S7, and S11 Files, respectively. IPA, Ingenuity Pathway Analysis; mNP, match NP; NLR, nonlinear regression; SZ, schizophrenia; ZT, Zeitgeiber time.

The SZ and mNP cohorts had similar timing patterns for transcripts with 12 h rhythms, with one population of transcripts that peaked in expression in the morning/evening, and the other that peaked during the afternoon/night (Fig 5A and S1 File). However, IPA and Metascape analyses indicated that mitochondria, EIF2 signaling, and protein ubiquitination pathways were associated with morning/evening 12 h rhythmic transcripts in the mNP cohort, but with afternoon/night transcripts in the SZ cohort (Figs 5B, 5D, and S8A and S7 and S11 Files). Transcripts associated with the UPR also peaked during the morning/evening in the mNP cohort but, consistent with the loss of rhythmicity analysis, were not associated with either population in the SZ cohort (Fig 5B and S7 and S11 Files). Similarly, transcripts associated with the cytoskeleton and synaptogenesis (Synaptogenesis Signaling Pathway, Reelin Signaling in Neurons, Actin Cytoskeleton Signaling, RhoA Signaling) peaked in expression during the afternoon/night in the mNP cohort but were not associated with either time point in the SZ cohort (Figs 5B, 5C, and S8A and S7 and S11 Files). While most transcripts with 12 h rhythms peaked in the morning/evening in SZ, there was little distinct pathway recognition in either the IPA or Metascape analyses (S7 and S11 Files). In both analyses, histone/chromosome regulation (histone h3 k36, positive regulation heterochromatin, regulation transcription initiation) was identified as a top pathway/biological process, though at a notably lower level of enrichment than the other groups (Figs 5B, 5E, and S8B and S7 and S11 Files).

### Multiple peak time-dependent patterns of motif enrichment differences in SZ

Finally, we applied motif enrichment analysis to these groups (mNP morning/evening, mNP afternoon/night, SZ morning/evening, SZ afternoon/night) to determine whether there may be differences in upstream regulators associated with 12 h rhythms in SZ (S9 Fig and S8 File). Additionally, we split transcripts that significantly lost/gained 12 h rhythms by peak time and performed a motif enrichment analysis (S9 Fig and S8 File). We found that ETS domain, PAR bZIP, POU class homeobox, PRD class homeobox, Forkhead box, and SRY-box families were associated with motifs enriched in the mNP morning/evening group of transcripts, but none of these families were enriched in the SZ morning/evening group (S9A, S9B and S9D Fig). Consistent with this, these families were associated with the morning/evening peaking transcripts that lost rhythmicity (S9A, S9C, and S9E Fig). The ETS domain family, however, was enriched in the SZ afternoon/night group and in transcripts that gained rhythmicity in the afternoon/night, suggesting that, like the mitochondria pathways observed earlier, this protein family was associated with transcripts that have altered timing in SZ (S9A–S9C Fig). Similarly, the KLF, SP domain family, and BHLH domain family were associated with motifs enriched in transcripts that peak in the afternoon/night in the mNP cohort, but with the morning/evening group in the SZ cohort (S9A and S9F Fig). These distinct patterns in enrichment give us insight into which pathways may be regulating the multifaceted differences observed in 12 h rhythms in SZ.

## Discussion

Biological rhythms allow organisms ranging from bacteria to humans to anticipate changes in the environment across the light/dark cycle and adapt accordingly. These rhythms occur on many scales, from seasons to days to hours, and the importance of circadian rhythms in health and disease has become increasingly clear over the past few decades, particularly in the context of psychiatric illnesses [21,22]. Far less is known about ultradian rhythms, including how prevalent they are in the human brain, and whether they are disrupted in subjects with psychiatric disorders. In this study, we characterized the 12 h transcriptome within the DLPFC of NP and SZ subjects. These rhythms have distinct timing patterns, splitting them largely into two populations of transcripts. One of these peaks in the morning/evening (ZT 2 to 3/14 to 15; approximately 9 AM/PM) and is largely associated with mitochondria, EIF2 signaling, and the UPR. The other peaks during the afternoon/night (ZT 8 to 9/20 to 21; approximately 3 AM/PM) and is associated with cytoskeleton dynamics and the processes necessary to build and maintain neuronal connections.

These findings indicate that 12 h rhythms in the brain are associated with processes necessary for essential cellular functions—and may be fundamental for timing processes to maximize resources and reserve energy when not needed. This is consistent with previous analyses in mouse liver, which uncovered 12 h rhythms in metabolism-related transcripts and processes fundamental to transcription, RNA splicing, translation, and proteostasis [23,24]. An exploratory analysis comparing 12 h rhythms in the mouse liver to our findings identified 620 overlapping genes (S5 Fig and S14 File), suggesting a high degree of similarity between the two studies despite differences in technique, species, and region [25]. In the liver, the timing of these processes coincides with sleep/wake transition times, leading to the proposal of a vehicle-cargo hypothesis for 12 h rhythms, in which 12 h rhythmicity accommodates increased demand for gene expression/processing at biological “rush hours” (i.e., sleep/wake transitions) by up-regulating expression of factors that facilitate protein and energy production [23]. Our observation that mitochondria- and translation-associated transcripts in human brain peak in expression in the morning/evening—when individuals are likely transitioning from wake to sleep or sleep to wake—strongly supports this hypothesis.

Strikingly similar to the mouse liver, our motif enrichment analysis implicated the ETS domain, KLF, and SP domain families in ultradian rhythm regulation (Fig 2A). Individual members of these transcription factor families also had significant 12 h rhythms, marking them as potential transcription factor regulators of these rhythms (Fig 2B). Consistent with our pathway analyses, separation of the data into two populations by peak time resulted in increased clarity. We found that transcripts that peak in the morning/evening are regulated by the POU class of homeoboxes and PAR bZIP family, while transcripts that peak in the afternoon/night are regulated by KLF, SP domain, and BHLH domain families (Fig 2C). The PAR bZIP and BHLH families are both implicated in circadian rhythms [15], suggesting that at least some proportion of what we have identified as 12 h rhythms could be regulated by the canonical circadian clock; however, the BHLH family is very large and the circadian clock regulators are not the top hits within this group.

We observed far fewer transcripts with 12 h rhythms in SZ subjects than in the mNP subjects. Intriguingly, the UPR is the top pathway associated with transcripts that no longer have 12 h rhythms in SZ. The UPR has been shown to regulate 12 h rhythms in in vivo culture models and mouse liver [23,25]. Consistent with this, differential expression of UPR proteins and markers of UPR activity have been found in the DLPFC of subjects with SZ [26]. SZ subjects also do not display rhythmicity in transcripts associated with the actin cytoskeleton and synaptogenesis, which peak during the afternoon/evening in the mNP subjects (Fig 5). Genomic analyses strongly implicate synaptogenesis and synaptic plasticity processes, while neuroanatomical studies have demonstrated reduced dendritic spine density in various regions, including the DLPFC, in SZ [27,28]. Of particular note, several voltage-gated calcium channels (CACNAs), a couple of which are top SZ risk factor genes [27], are among this group with 12 h rhythms in mNP subjects but no rhythms in SZ.

The NLR analysis showed relatively little direct overlap in the transcripts that are identified as rhythmic in the mNP and SZ cohorts (Fig 3 and S1 File). However, a threshold-free approach (S6 Fig) and pathway analyses indicate some degree of overlap. This is best exemplified by 12 h rhythms in mitochondria-associated pathways, which are not lost but instead switch from peaking during the morning/evening to afternoon/night time points in SZ. This could have profound effects on mitochondrial energy production at times of day when this is most needed. Various studies have implicated circadian rhythms in mitochondria biology, including gene expression in the mouse SCN, oxygen consumption and mitochondria respiration in isolated mitochondria from rat brains, and fusion/fission states in mouse macrophages [29–31]. Interestingly, mitochondrial-related pathways also gained 24 h rhythms in the DLPFC of subjects with SZ [8] (Fig 4), resulting in a convergence of rhythmicity dysregulation in mitochondrial function transcript expression. Gaining a 24 h rhythm may be a compensatory measure to account for suboptimal timing of 12 h rhythms or reflect changes associated with diminished neuronal activity specifically at night. Our findings are consistent with a robust literature of mitochondrial abnormalities in SZ, from genetics to function, number, location, and shape [32]. It will be interesting in future studies to determine changes in mitochondrial morphology, number, function, and location in subjects with SZ as a function of time of day, and how this relates to transcript expression.

This study’s findings fit into a growing body of literature attempting to characterize mammalian 12 h rhythms. 12 h rhythms were first observed as circatidal rhythms, in which coastal and estuarine animals show behavior aligned with the approximately 12.4 h ebb and flow of tides [23]. As researchers studied these behaviors, two theories on the molecular mechanisms emerged: The first suggested that there are two circadian clocks acting in antiphase to produce the 12 h peaks in behavior, while the second proposed a dedicated 12 h molecular clock. Several studies have reported that disrupting light and the circadian clock does not impact circatidal rhythms, providing support for the second hypothesis [1]. The study of 12 h rhythms in mammals, is much newer, with the first study showing 12 h rhythms in transcript expression in mouse liver and other tissues occurring in 2009 [33]. Multiple studies have since confirmed the existence of 12 h rhythms in mouse liver [25,33–36]. The same debate over the molecular mechanism that generates these rhythms has occurred within this group, with some evidence for two circadian antiphasic regulators [33,36] and others demonstrating that 12 h rhythms are dependent on the UPR and demonstrating involvement of specific transcription factors [35,37,38]. Our findings best align with the idea of a dedicated 12 h clock, but future work in cell and animal models will be necessary to confirm this.

The superchiasmatic nucleus (SCN) is the master pacemaker of circadian rhythms in the brain, but whether ultradian rhythms are regulated across regions by a dedicated system is unknown. While not specific to 12 h rhythms, levels of dopamine in the striatum fluctuate in synchrony with ultradian locomotor activity cycles and dopaminergic transmission directly regulates ultradian cycle length in mice [39]. Interestingly, these dopamine-driven ultradian cycles in locomotor activity harmonize with circadian rhythms coordinated by the SCN, but if this relationship is disrupted, it can lead to altered patterns of arousal and disrupted sleep/wake cycles when desynchronized [39], which are commonly observed in subjects with SZ. SZ has long been associated with altered dopaminergic transmission in cortico-striatal pathways [40]. Abnormalities in dopaminergic signaling, therefore, may be a potential mechanism by which ultradian rhythms are disrupted across the brain in SZ.

Due to the limited sample size and novel nature of the study design, many of the analyses in our study are exploratory and use *p*-value cutoffs for determining statistical significance. As such, we chose to focus throughout on the overall patterns and themes that the study illuminated, rather than individual genes identified as rhythmic. Additionally, human postmortem brain tissue research presents a variety of limitations. We have addressed several through our study design, which includes exclusion of subjects older than 65, strict TOD criteria, and cohorts matched for a number of important biological, clinical, and technical factors. However, factors like disrupted sleep, antipsychotic medication, and nicotine use are common in the SZ cohort, with low to no prevalence in the NP cohort. As such, we are unable to determine whether our findings are a component of SZ pathophysiology, due to one of these factors, or an interaction between the two.

Sleep disturbances are frequently observed in patients with SZ, with prevalence rates reported to be approximately 80% [41,42]. Higher rates of sleep disruption, particularly insomnia, are noted in both chronic and first-episode populations [42]. Additionally, sleep disruption is associated with symptom severity of positive and cognitive symptoms [42]. Intriguingly, sleep disturbances often precede episodes of psychosis and/or worsening of symptoms and have been observed in young populations at high risk for psychosis [42]. Similar to other psychiatric illnesses like bipolar disorder and major depression, sleep architecture abnormalities like reduced slow-wave sleep, increased sleep onset latency, decreased total sleep time, and decreased sleep efficiency have been repeatedly shown in subjects with SZ [42]. Notably, SZ patients also have decreased sleep spindle density, which is not found in other psychiatric populations [42], suggesting that there may be a unique relationship between SZ and sleep. While we are not looking directly at sleep in this study, altered/sleep wake cycles can influence molecular rhythms [43–45], and as such, we do not know whether our findings are due to the sleep disturbances experienced by these patients, a component of the disease pathology, or a combination of the two. While the TOD information for these patients has allowed us to perform the analyses we report here, we do not have information on each subject’s sleep patterns, which may be important for interpreting our findings. Future work, using both animal and human postmortem models, defining how chronotype and sleep disruptions impact the 24 and 12 h transcriptomes in the prefrontal cortex will be necessary to begin teasing apart how these dynamics impact molecular rhythms in SZ.

Antipsychotic medications are also important to consider, as the majority of the SZ cohort is on them while none of the NP cohort is. Antipsychotic medications could be having an impact from multiple directions, as they can directly impact gene expression as well as improve sleep in some patients [41,46,47]. Antipsychotics can improve total sleep time and efficiency, though this is variable dependent on the type of medication, with atypical antipsychotics being more effective than typical [41]. A few studies have attempted to look at the impact of antipsychotics on circadian gene expression in rodents but have had mixed results [41]. Future work in populations taking antipsychotics that are not diagnosed with psychoses and in rodent models will be necessary to determine what impact antipsychotics are having on gene expression, and whether this is indirectly through their sedative properties.

In contrast, SZ subjects use nicotine, which has stimulant properties, at much higher rates than the general population [48]. This use typically starts before disease onset, and higher smoking rates are associated with poorer quality of life, worse prognosis, and increased disease severity [48]. It is possible that the disproportionate nicotine use and disruptions in the cholinergic system may be contributing to our findings. This could be through many mechanisms, as this system is incredibly complex and heavily interwoven into both cognition and sleep [49].

In conclusion, to our knowledge, this study is the first to identify 12 h rhythms in transcript expression in the human brain. These rhythms are associated with fundamental cellular processes. However, in SZ, there is a strong reduction in the number of transcripts with 12 h rhythms, along with altered timing of transcripts important in mitochondrial function. Future studies will determine the functional consequences of these findings to optimal brain health and the pathophysiology of brain disorders.

## Materials and methods

### Human postmortem brain samples

Human postmortem RNA-seq data for 613 samples were obtained from the CommonMind Consortium (https://nimhgenetics.org/available_data/commonmind/) and filtered based off previously described criteria [8,10]. Briefly, subjects were included if they met the criteria of rapid death (<2 h elapsed time between precipitating event and death announcement) and had a postmortem interval (PMI) of <30 h. In addition, subjects with age >65 years were removed as our lab has previously observed significant differences in molecular rhythms between young and elderly subjects [6]. A total of 104 NP subjects and 46 subjects with SZ met these criteria (S1 Table). The larger sample size of NP subjects may result in more statistical power for rhythm detection. Therefore, 46 NP subjects that were best matched to the 46 SZ subjects by age, sex, race, TOD, PMI, site of collection, and pH (mNP cohort; S1 Table). The subjects in these cohorts are the same as those used in a previous study performed by our lab focused on circadian rhythms [8].

### Time of death analysis in the Zeitgeber time scale

Prior to rhythmicity analysis, TOD for each subject was normalized to a ZT scale as described in Seney and colleagues [8]. Briefly, the TOD for each subject was collected at local time then converted to coordinated universal time by adjusting time zone and daylight savings time. Coordinated universal time was further adjusted to account for longitude, latitude, and elevation of death place, and each subject’s TOD was set as ZT = *t* h after previous (if *t* < 18) or before next (if *t* ≥ −6) sunrise. The distribution of subject TODs is shown in S1 Fig.

### RNA sequencing data preprocessing

Samples were analyzed using RNA-seq and 30,714 unique genes were identified. Genes were retained for analysis if counts per million (cpm) was greater than 1 in 50% of more subjects. All Y chromosome genes were eliminated along with transcripts with no identifiers. After filtering, 13,914 genes remained, and expression of these genes were log2 normalized. Since samples in the CommonMind dataset were generated in 2 brain banks, we included equal proportions of Pittsburgh and Mt. Sinai individuals in each experimental group and used the ComBat function of the SVA R package [50] to perform site correction of the normalized and filtered data.

### Rhythmicity analyses

#### Nonlinear regression analysis

We utilized a modified version of the sinusoidal NLR analysis that has been used multiple times to detect circadian patterns of gene expression in human postmortem brain tissue (by our group and others) [5–9]. While previous papers defined the period of the sinusoidal curve as 24 h, we modified this analysis to detect genes that best fit a sinusoidal curve with a 12 h period. *P* values were obtained by comparing the observed coefficient of determination (R^2^) and a null distribution R^2^ that was generated by fitting the sinusoidal curve to 1,000 TOD-randomized data points. The results of this analysis can be found in S1 File. We confirmed the robustness of our analysis through bootstrapping (S2 Fig and S2, S9, and S10 Files). In our bootstrapping analyses, we resampled from each cohort 100 times.

#### Lomb–Scargle analysis

We performed a second analysis, the Lomb–Scargle periodogram, through the R package MetaCycle [13,51]. Lomb–Scargle is a method of rhythm detection primarily used in physics. It is unique in its capacity to analyze unevenly sampled time series data, to identify rhythmic expression profiles with a range of periods, and to differentiate between periodic and nonperiodic profiles for cosine curves with high noise [13]. In this study, we analyzed the data for rhythms with periods between 8 and 28 h. These periods were chosen after testing several ranges and finding that there is an artificial buildup of rhythms with their period identified as the period boundary. 8 and 28 h appeared sufficiently far away from 12 and 24 h such that the shape of these transcript populations were unaffected by this artificial buildup. It is important to note when interpreting these results, that to get a true representation of transcripts with 24 h rhythms, we would have needed to use data with a 48 h TOD range. The results of this analysis can be found in S3 File.

#### Eigenvalue/Pencil analysis

Prior to analysis, weighted averages of every transcript were calculated for each hour interval for a range of ZT −5 to 42 (S4 Fig). Samples were then analyzed using the eigenvalue/pencil method as previously described [19,25]. This method assumes gene expression is the result of multiple superimposed oscillations and identifies the combination of RCs that best explains the data without any constraints on the period, amplitude, or phase of the rhythms. The eigenvalue/pencil method was set to identify the top 4 RCs contributing to gene expression. We used the eigenvalue/pencil method to identify four superimposed RCs that best explain transcript expression for all 13,915 transcripts identified through RNA-seq in the DLPFC of NP and SZ subjects (S1 Fig). The period of these RCs varied between 2 and Infinity (S4 Fig). The sampling rate of our cohorts, and the way in which we preprocessed data before applying the eigenvalue/pencil method, led us to consider periods below 9 or above 30 as indistinguishable from noise. The results of this analysis can be found in S4, S5, S12, and S13 Files.

### Pathway enrichment and upstream regulator analyses

#### Ingenuity pathway analysis

Pathway and upstream regulator analyses were performed on transcript lists based on either the period (eigenvalue/pencil) or the *p*-value of the respective rhythmicity analysis. A user-provided list of the 13,915 transcripts expressed in the DLPFC was used as the background. A total of 13,454 background transcripts remained, as some of the transcripts were not mapped or functionally annotated. Pathways and upstream regulators were considered enriched if they met a threshold of *p* < 0.05 (-log_10_(*p*-value) > 1.3 in figures). IPA pathways with <15 or >300 genes were not included in the analyses, and only direct relationships were considered for the upstream regulator analysis. The top 5 significant pathways and upstream regulators are represented for each analysis. Complete lists of all significant pathways and upstream regulators for each analysis can be found in S7 File.

#### Biological process enrichment

The web-based portal Metascape was used to perform biological process enrichment (May 2021) [18]. Process enrichment was accomplished using GO biological processes as the ontology source. Within Metascape, the multigene list meta-analysis option was used to compare process enrichment between 12 and 24 h RCs or expression rhythms, diagnosis, and/or analysis method. Similar to IPA, transcript lists were based on either the period or the *p*-value (*p* < 0.05) of the respective rhythmicity analysis. However, Metascape analysis limits the number of transcripts to ≤3,000. Only the eigenvalue/pencil analysis lists were longer than 3,000 transcripts, so we used the top 3,000 transcripts based on the largest amplitude values of the RCs. A user-supplied list of the 13,915 transcripts expressed in the DLPFC was used as the enrichment background. A total of 13,521 background transcripts remained, as some of the transcripts were not mapped or functionally annotated. Terms with *p* < 0.01, a minimum count of 3, and an enrichment factor of >1.5 were grouped into clusters based on their membership similarities (kappa score > 0.3). *P* values were calculated based on the cumulative hypergeometric distribution, and q-values were calculated based on the Benjamini–Hochberg procedure [18]. The most statistically significant term within a cluster was chosen to represent the cluster. The top 20 significant clusters of processes are depicted by Cytoscape [20]. If more than 10 enriched terms within a cluster were identified, the top 10 significant terms were chosen for visualization in the Cytoscape networks. Complete lists of all the enriched processes within each cluster can be found in S11 File. In the network plots, the nodes are represented as pie charts, where the size of the pie is proportional to the total number of gene hits for that specific term. The pie charts are color-coded based on the identity of the gene list, where the size of the slice represents the percentage of genes for the term that originated from the corresponding list. Terms that are similar (kappa score > 0.3) are connected by edges.

### LISA Cistrome combined motif enrichment analysis

The web-based LISA program was used to perform motif enrichment analyses [14]. For supplied gene lists, LISA determined whether a set of known motifs were enriched. The combined enrichment scores (-log10(pvalue)) were used to interpret results and can be found in S8 File.

### Data visualization

#### Scatterplots

We generated scatterplots of individual transcript expression for the top three transcripts with 12 h rhythms in expression. Each dot represents a subject with the *x* axis indicating the TOD on ZT scale and *y* axis indicating gene expression level after log2 normalization. The line superimposed on the graph is the fitted sinusoidal curve (Fig 4A–4C). Data used in scatterplots are provided in S6 File.

#### Heatmaps

Heatmaps for transcripts transcript expression across TOD were generated for the top 100 genes with significant 12 h rhythms in the fNP cohort (*p* < 0.01; Fig 1A) and for mitochondria electron transport chain complex genes in both the mNP and SZ cohorts (Fig 3E and 3F) using the matrix visualization and analysis software Morpheus (https://software.broadinstitute.org/morpheus). Expression levels were *Z-*transformed for each transcript, and transcripts were ordered by the time at which they peak. Subjects were ordered by their TOD and grouped by ZT hour. Each column is the median gene expression for subjects within each ZT hour group.

### Rank-Rank Hypergeometric Overlap (RRHO)

RRHO is a threshold-free approach that identifies the overlap between two lists of transcripts ranked by their log_10_(*p*-value) [17] as determined through NLR (S1 File). This approach avoids an arbitrary threshold in conventional Venn diagram approaches.

## Supporting information

S1 Fig TOD distributions. TOD values for subjects in the (**A**-**B**) fNP (*n =* 104), (**C**-**D**) mNP (*n* = 46; mNP), and (**E**-**F**) SZ (*n* = 46) cohorts plotted as frequency distributions (**A**, **C**, **E**) and around a 24 h circle plot (**B**, **D**, **F**). fNP, full NP; mNP, match NP; SZ, schizophrenia; TOD, time of death.
(TIFF)

S2 Fig Bootstrap analysis on NLR rhythmicity analysis. (**A**-**C**) Bootstrapping was performed by resampling from each cohort 100 times. The number of times each transcript identified in the original analysis was identified as significantly rhythmic in the bootstrap analyses is shown, (**A**) with *p* < 0.01 used as the threshold for the fNP cohort and (**B**-**C**) *p* < 0.05 used as the threshold for the (**B**) mNP and (**C**) SZ cohorts. The results of the bootstrapping analysis can be found in S2, S9, and S10 Files. fNP, full NP; mNP, match NP; NLR, nonlinear regression; SZ, schizophrenia.
(TIFF)

S3 Fig Additional rhythmicity analyses demonstrating 12 h rhythms in human DLPFC. (**A**-**B**) Lomb–Scargle analysis. Points above the red dotted lines have *p*-values < 0.05 in the Lomb–Scargle analysis, while black dotted lines indicate a region of period enrichment between 10–12 h. (**A**) Periods and *p*-values of all transcripts, *p* < 0.05 is indicated in blue. (**B**) The periods and *p*-values determined by the Lomb–Scargle analysis of transcripts identified as having a significant (*p* < 0.01) 12 h rhythm by the NLR analysis. Results of the Lomb–Scargle analysis can be found in S3 File. (**C**-**E**) An abbreviated example of the eigenvalue/pencil method for a single gene is shown, with the (**C**) initial expression values, the (**D**) RCs reported by the analysis, and (**E**) a graphical representation of the RCs superimposed on each other. (**F**-**G**) The eigenvalue/pencil analysis in human DLPFC. (**F**) A histogram of RC periods, with the range of what we considered to be 12 h RCs highlighted in blue. Results from the eigenvalue/pencil analysis are presented in S4 File. 12 and 24 h RCs identified are shown in S5 File. (**G**) The top 5 pathways identified by an IPA of transcripts with 12 h rhythms (blue) and 12 h RCs (white). IPA results can be found in S7 File. DLPFC, dorsolateral prefrontal cortex; IPA, Ingenuity Pathway Analysis; NLR, nonlinear regression; RC, rhythmic component.
(TIFF)

S4 Fig Applying the eigenvalue/pencil analysis to human postmortem brain tissue. Example of applying the eigenvalue/pencil method to RNA-seq data from human postmortem brain tissue. (**A**) Example of gene expression across TOD. (**B**-**D**) Preprocessing steps to convert to even-interval data. (**E**-**F**) Example of eigenvalue/pencil results for an individual gene. (**G**) Histogram of RC periods from all 13,915 genes analyzed. Raw output from the eigenvalue/pencil analysis can be found in S4, S12, and S13 Files for the fNP, mNP, and SZ cohorts, respectively. Results summarizing the 12 and 24 h RCs of the eigenvalue/pencil analysis can be found in S5 File. fNP, full NP; mNP, match NP; RC, rhythmic component; RNA-seq, RNA sequencing; SZ, schizophrenia; TOD, time of death.
(TIFF)

S5 Fig Comparison of genes identified as having 12 h rhythms in human DLPFC and mouse liver. (**A**-**B**) Heatmaps of the 620 genes that were identified as having 12 h rhythms in both (**A**) human DLPFC and (**B**) mouse liver. Subjects are ordered by ZT (h) on the *x* axis. ZT = 0 is (**A**) sunrise in the human subjects and (**B**) 7:00 AM on the first day of the experiment in mice. Details of data reported in this figure can be found in S14 File. DLPFC, dorsolateral prefrontal cortex; ZT, Zeitgeiber time.
(TIFF)

S6 Fig RRHO comparison of 12 h rhythmicity between cohorts. RRHO plots comparing the (**A**) full and match NP cohorts, the (**B**) fNP and SZ cohorts, and the (**C**) mNP and SZ cohorts. (**D**-**F**) Corresponding Rank-Rank scatterplots for each cohort comparison are shown. These show each genes *p*-value rank within each cohort compared to the other cohorts. (**G**-**I**) Scatterplots comparing the original -log(*p*-values) for each gene between cohorts. These plots were created with information that can be found in S1 File. fNP, full NP; mNP, match NP; NP, nonpsychiatric; RRHO, rank-rank hypergeometric overlap; SZ, schizophrenia.
(TIFF)

S7 Fig Biological processes enriched for transcripts with 12 and 24 h rhythms in expression. Cytoscape depiction of a Metascape analysis of transcripts with 12 and 24 h rhythms in both the mNP and SZ cohorts. Metascape results can be found in S11 File. mNP, match NP; SZ, schizophrenia.
(TIFF)

S8 Fig Biological processes enriched in transcripts with 12 h rhythms that peak either in the morning/evening or in the afternoon/night. (**A**) Heatmap of Metascape analysis of transcripts with 12 h rhythms that peak in expression either in the ME or AN. (**B**) Biological processes enriched in the SZ ME group analyzed separately due to much lower strength of enrichment than the other 3 groups. Metascape results can be found in S11 File. AN, afternoon/night; ME, morning/evening; SZ, schizophrenia.
(TIFF)

S9 Fig Time-dependent differences in motif enrichment between mNP and SZ cohorts. (**A**) Summary of patterns in protein families associated that emerge after motif enrichment analysis. Families include ETS domain family (ETS), PAR bZIP family, POU class homeoboxes, PRD class homeoboxes, Forkhead boxes, SRY boxes, KLFs, SP domain family (SP), BHLH domain family (BHLH). (**B**, **D**, **F**) Comparison of motif enrichment scores (-log10(pvalue) between mNP and SZ for transcripts that peak either in the ME or AN. (**C**, **E**, **G**) Comparison of motif enrichment scores between ME and AN groups for transcripts that either significantly lose 12 h rhythms in SZ or significantly gain 12 h rhythms in SZ. (**B**-**C**) ETS domain family, which shows altered timing in SZ (mNP ME to SZ AN). (**D**-**E**) Examples of protein families have enrichment in mNP ME, but no enrichment in any SZ groups. (**F**-**G**) Examples of protein families with altered timing in SZ (mNP AN to SZ ME). Plots in this figure were created from data that can be found in S8 File. AN, afternoon/night; BHLH, Basic Helix–Loop–Helix; bZIP, basic leucine zipper; KLF, Kruppel-like factor; ME, morning/evening; mNP, match NP; SRY, Sex determining region Y; SZ, schizophrenia.
(TIFF)

S1 Table Description of CommonMind Consortium cohorts. (PDF)

S2 Table Number of transcripts with significant 12 or 24 h rhythms identified by a sinusoidal nonlinear regression. (PDF)

S1 File Sinusoidal NLR analyses. (Tabs 1–3) Results of the sinusoidal NLR analyses identifying 12 and 24 h rhythms. (Tab 4) 12 h rhythm parameter comparisons between mNP and SZ cohorts, including gain/loss analysis. mNP, match NP; NLR, nonlinear regression; SZ, schizophrenia.
(XLSX)

S2 File Bootstrapping analysis of 12 h rhythmicity in human DLPFC. Bootstrapping was performed by resampling from the fNP cohort 100 times. The *p*-values obtained for each gene (rows) are shown for every sampling attempt (columns). DLPFC, dorsolateral prefrontal cortex; fNP, full NP.
(CSV)

S3 File Lomb–Scargle analysis. (Tab 1) Results of the Lomb–Scargle analysis for all genes. (Tab 2) Abbreviated results of the Lomb–Scargle and NLR analyses for genes with significant 12 h rhythms in the NLR analysis (*p* < 0.01). NLR, nonlinear regression.
(XLSX)

S4 File Eigenvalue/pencil analysis of human DLPFC. Raw output of the eigenvalue/pencil analysis of the fNP cohort. DLPFC, dorsolateral prefrontal cortex; fNP, full NP.
(XLSX)

S5 File 12 and 24 h RCs in NP and SZ DLPFC. Results of the eigenvalue/pencil analysis. (Tab 1) 12 h RCs (11 ≤ Period < 13). (Tab 2) 24 h RCs (20 ≤ Period < 26). (Tab 3) Genes with both 12 and 24 h RCs. DLPFC, dorsolateral prefrontal cortex; NP, nonpsychiatric; RC, rhythmic component; SZ, schizophrenia.
(XLSX)

S6 File Gene expression scatterplots. Gene expression and TOD values used for scatterplots is provided here for the (Tab 1) fNP, (Tab 2) mNP, and (Tab 3) SZ cohorts. fNP, full NP; mNP, match NP; SZ, schizophrenia; TOD, time of death.
(XLSX)

S7 File Ingenuity Pathway Analyses. Ingenuity Pathway Analyses results for all groups. Each tab represents a different analysis.
(XLSX)

S8 File LISA analyses. (Tab 1) *P* values for all motifs analyzed in LISA Combined Cistrome Motif Enrichment Analysis. (Tab 2) *P* values only for motifs of genes expressed in our dataset. HGNC protein family category is listed.
(XLSX)

S9 File Bootstrapping analysis of 12 h rhythmicity in mNP Subjects. Bootstrapping was performed by resampling from the mNP cohort 100 times. The *p*-values obtained for each gene (rows) are shown for every sampling attempt (columns). mNP, match NP.
(CSV)

S10 File Bootstrapping analysis of 12 h rhythmicity in SZ Subjects. Bootstrapping was performed by resampling from the SZ cohort 100 times. The *p*-values obtained for each gene (rows) are shown for every sampling attempt (columns). SZ, schizophrenia.
(CSV)

S11 File Metascape analyses. (Tab 1–2) Results of Metascape analysis of 12 and 24 h rhythms from mNP and SZ cohorts. (Tab 1) Top 100 enriched biological pathways. (Tab 2) Full GO list and membership results used to create cytoscape images. (Tab 3–4) Results of Metascape analysis of 12 h rhythms that either peak during the morning/evening or afternoon/night in the mNP and SZ cohorts. mNP, match NP; SZ, schizophrenia.
(XLSX)

S12 File Eigenvalue/pencil analysis of mNP DLPFC. Raw output of the eigenvalue/pencil analysis of the mNP cohort. DLPFC, dorsolateral prefrontal cortex; mNP, match NP.
(XLSX)

S13 File Eigenvalue/pencil analysis of SZ DLPFC. Raw output of the eigenvalue/pencil analysis of the SZ cohort. DLPFC, dorsolateral prefrontal cortex; SZ, schizophrenia.
(XLSX)

S14 File Shared human and mouse 12 h rhythmic genes. List of 620 genes found to have significant 12 h rhythms in both the human DLPFC and mouse liver. The peak time in both datasets is given for each gene. DLPFC, dorsolateral prefrontal cortex.
(XLSX)
