# Supplementary figures and images for: Twelve-hour rhythms in transcript expression within the human dorsolateral prefrontal cortex are altered in schizophrenia
Source: PLoS Biol. 2023 Jan 24;21(1):e3001688. doi: 10.1371/journal.pbio.3001688 (PMC9873190; doi:10.1371/journal.pbio.3001688)

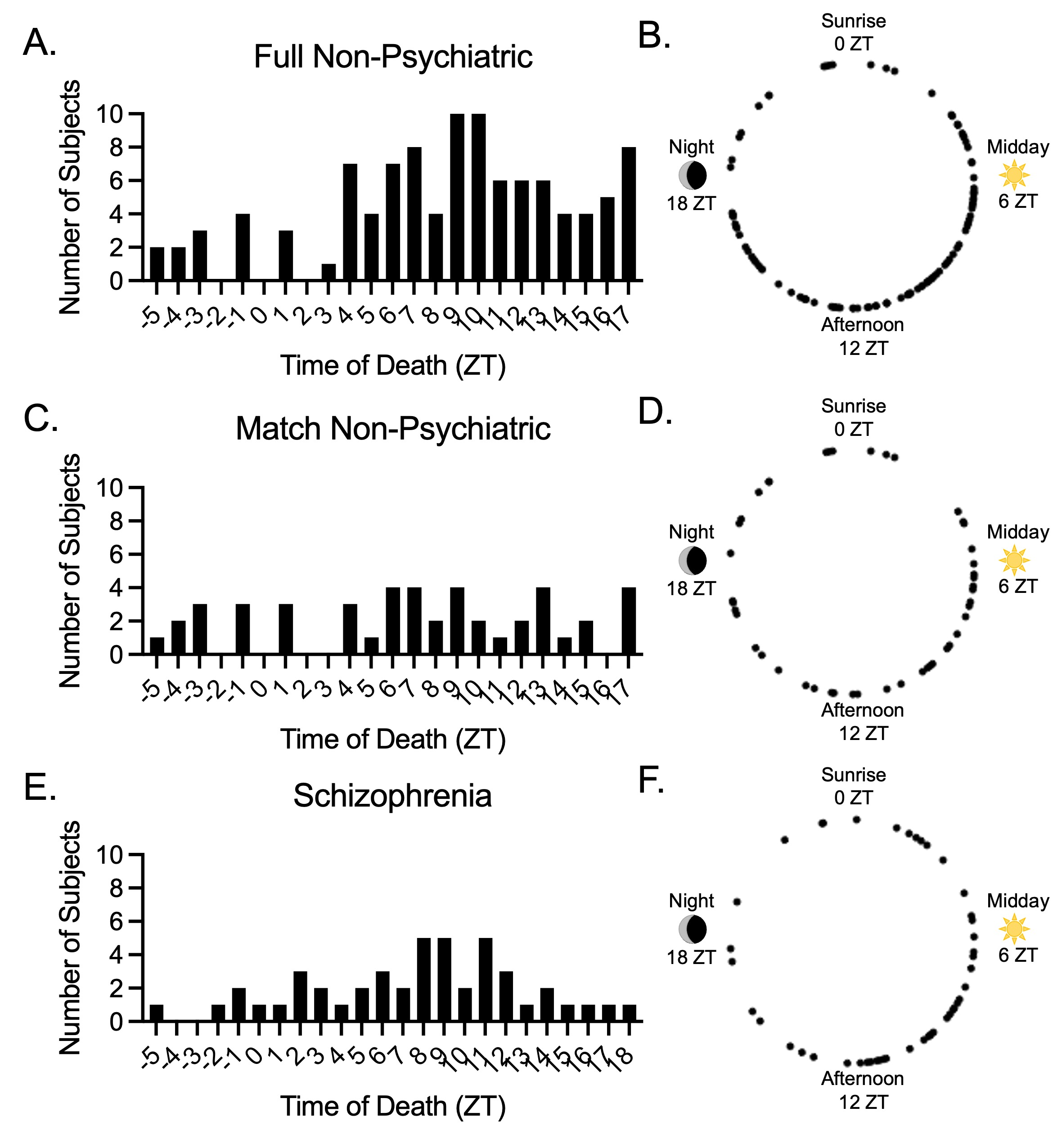

Supplement: S1 Fig — TOD values for subjects in the (A-B) fNP (n = 104), (C-D) mNP (n = 46; mNP), and (E-F) SZ (n = 46) cohorts plotted as frequency distributions (A, C, E) and around a 24 h circle plot (B, D, F). fNP, full NP; mNP, match NP; SZ, schizophrenia; TOD, time of death. (TIFF) [file pbio.3001688.s001.tiff]

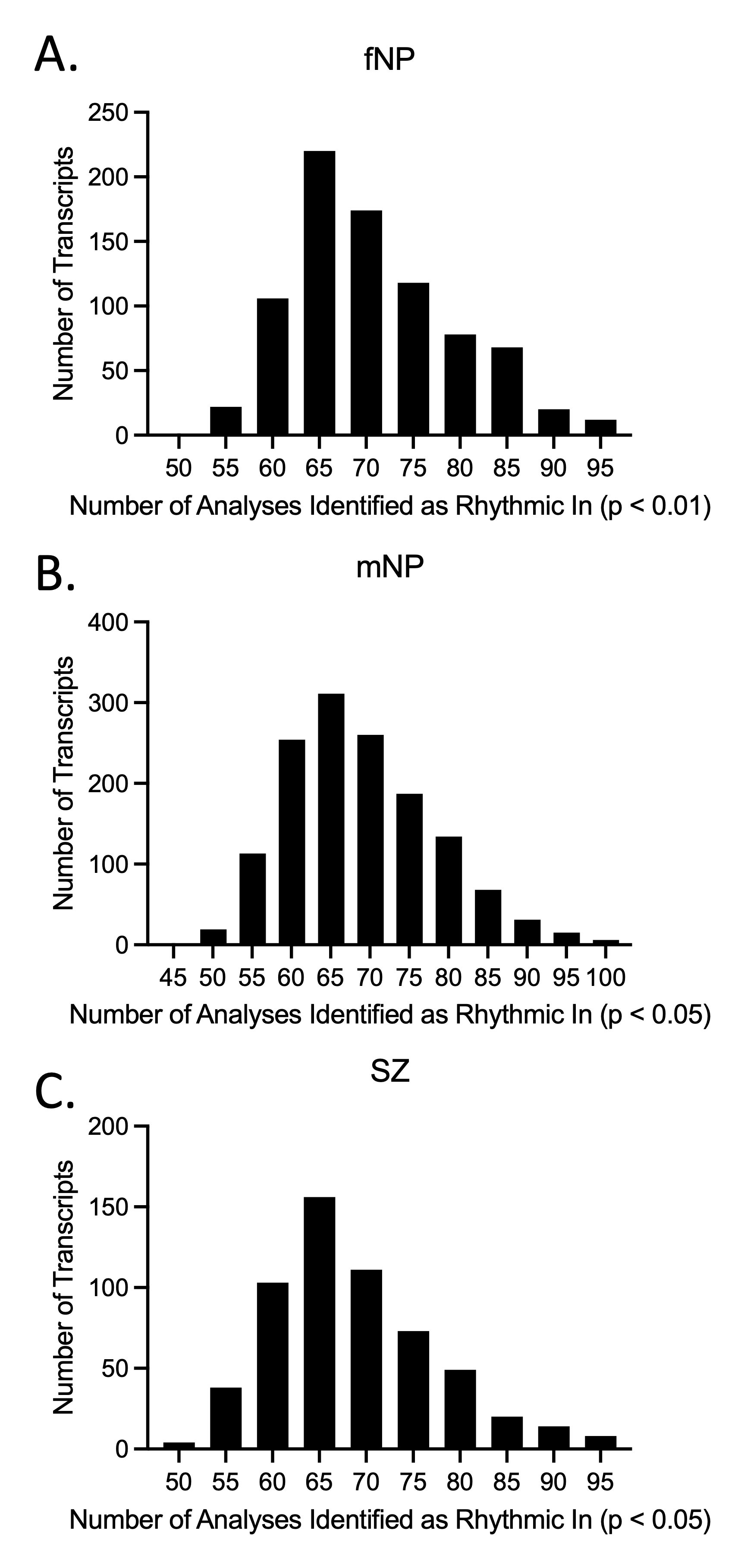

Supplement: S2 Fig — (A-C) Bootstrapping was performed by resampling from each cohort 100 times. The number of times each transcript identified in the original analysis was identified as significantly rhythmic in the bootstrap analyses is shown, (A) with p < 0.01 used as the threshold for the fNP cohort and (B-C) p < 0.05 used as the threshold for the (B) mNP and (C) SZ cohorts. The results of the bootstrapping analysis can be found in S2, S9, and S10 Files. fNP, full NP; mNP, match NP; NLR, nonlinear regression; SZ, schizophrenia. (TIFF) [file pbio.3001688.s002.tiff]

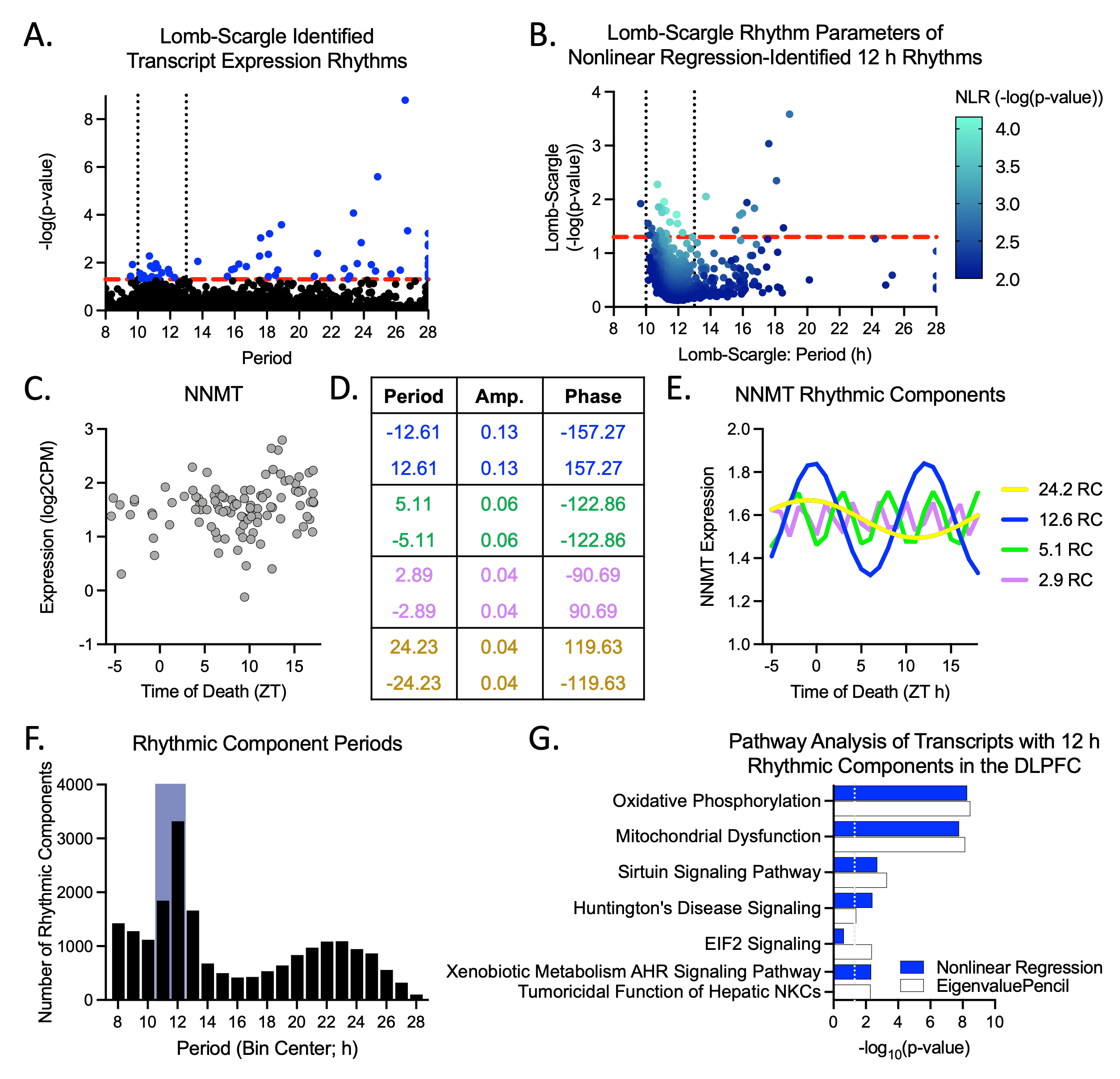

Supplement: S3 Fig — (A-B) Lomb–Scargle analysis. Points above the red dotted lines have p-values < 0.05 in the Lomb–Scargle analysis, while black dotted lines indicate a region of period enrichment between 10–12 h. (A) Periods and p-values of all transcripts, p < 0.05 is indicated in blue. (B) The periods and p-values determined by the Lomb–Scargle analysis of transcripts identified as having a significant (p < 0.01) 12 h rhythm by the NLR analysis. Results of the Lomb–Scargle analysis can be found in S3 File. (C-E) An abbreviated example of the eigenvalue/pencil method for a single gene is shown, with the (C) initial expression values, the (D) RCs reported by the analysis, and (E) a graphical representation of the RCs superimposed on each other. (F-G) The eigenvalue/pencil analysis in human DLPFC. (F) A histogram of RC periods, with the range of what we considered to be 12 h RCs highlighted in blue. Results from the eigenvalue/pencil analysis are presented in S4 File. 12 and 24 h RCs identified are shown in S5 File. (G) The top 5 pathways identified by an IPA of transcripts with 12 h rhythms (blue) and 12 h RCs (white). IPA results can be found in S7 File. DLPFC, dorsolateral prefrontal cortex; IPA, Ingenuity Pathway Analysis; NLR, nonlinear regression; RC, rhythmic component. (TIFF) [file pbio.3001688.s003.tiff]

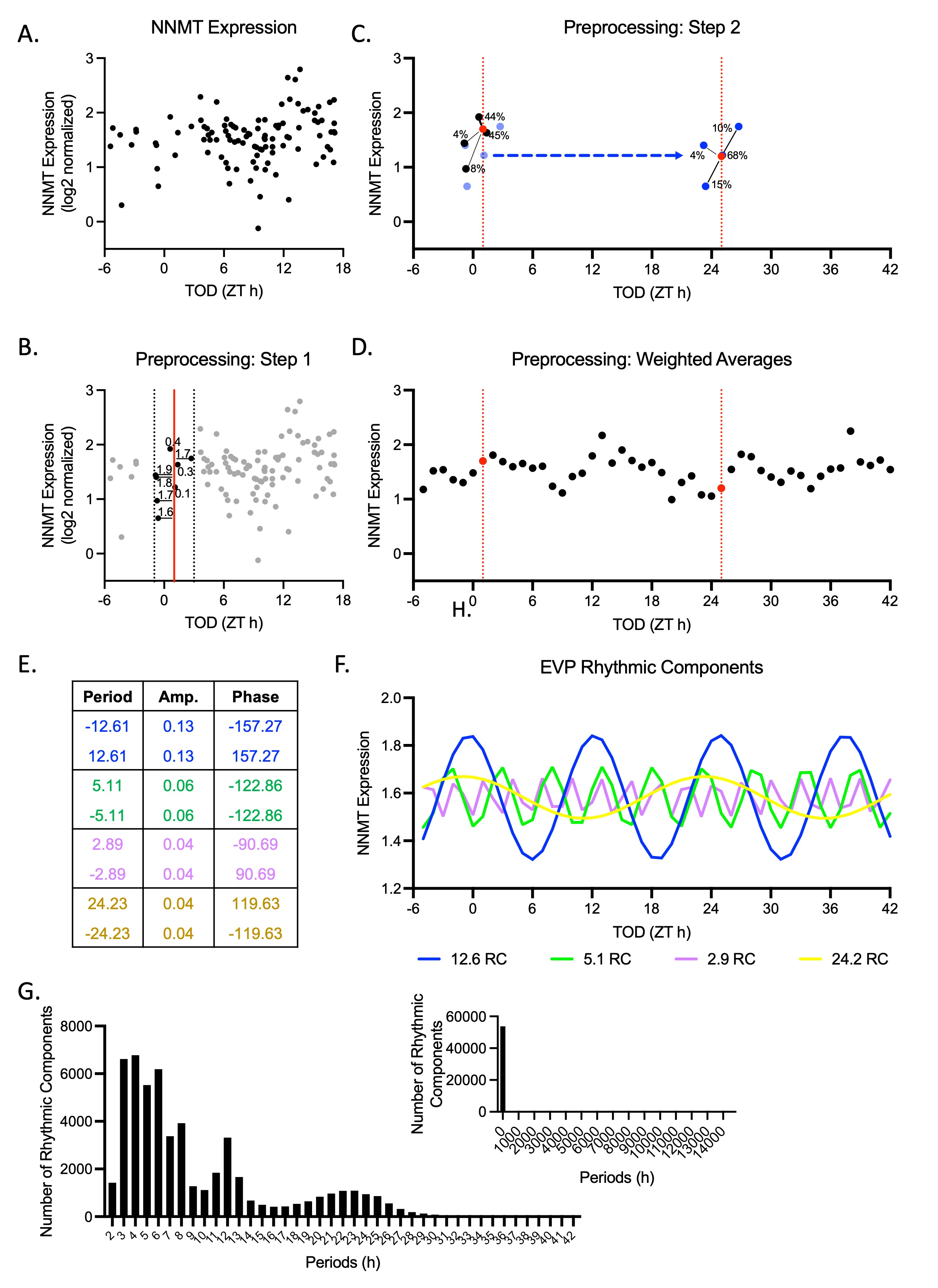

Supplement: S4 Fig — Example of applying the eigenvalue/pencil method to RNA-seq data from human postmortem brain tissue. (A) Example of gene expression across TOD. (B-D) Preprocessing steps to convert to even-interval data. (E-F) Example of eigenvalue/pencil results for an individual gene. (G) Histogram of RC periods from all 13,915 genes analyzed. Raw output from the eigenvalue/pencil analysis can be found in S4, S12, and S13 Files for the fNP, mNP, and SZ cohorts, respectively. Results summarizing the 12 and 24 h RCs of the eigenvalue/pencil analysis can be found in S5 File. fNP, full NP; mNP, match NP; RC, rhythmic component; RNA-seq, RNA sequencing; SZ, schizophrenia; TOD, time of death. (TIFF) [file pbio.3001688.s004.tiff]

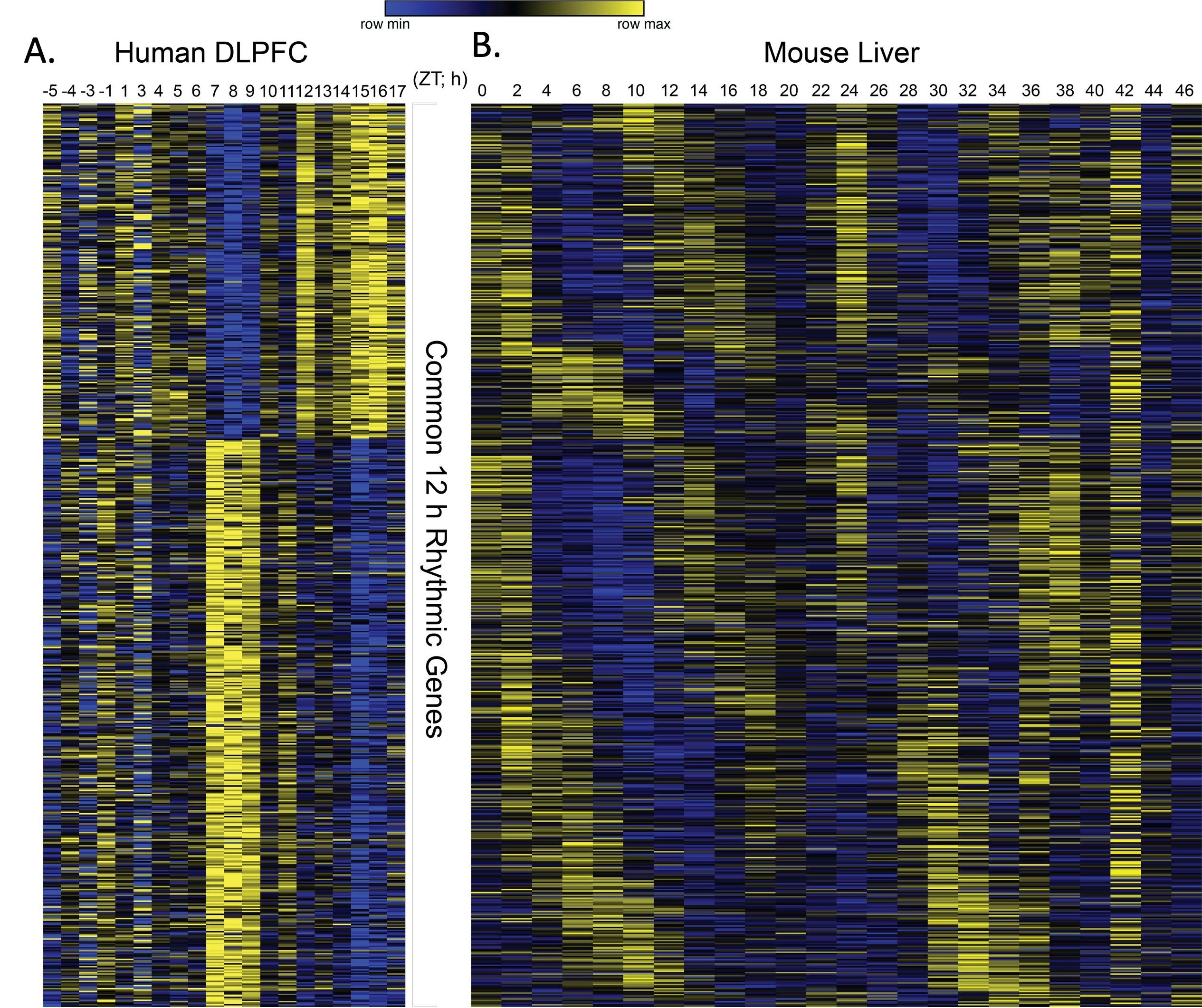

Supplement: S5 Fig — (A-B) Heatmaps of the 620 genes that were identified as having 12 h rhythms in both (A) human DLPFC and (B) mouse liver. Subjects are ordered by ZT (h) on the x axis. ZT = 0 is (A) sunrise in the human subjects and (B) 7:00 AM on the first day of the experiment in mice. Details of data reported in this figure can be found in S14 File. DLPFC, dorsolateral prefrontal cortex; ZT, Zeitgeiber time. (TIFF) [file pbio.3001688.s005.tiff]

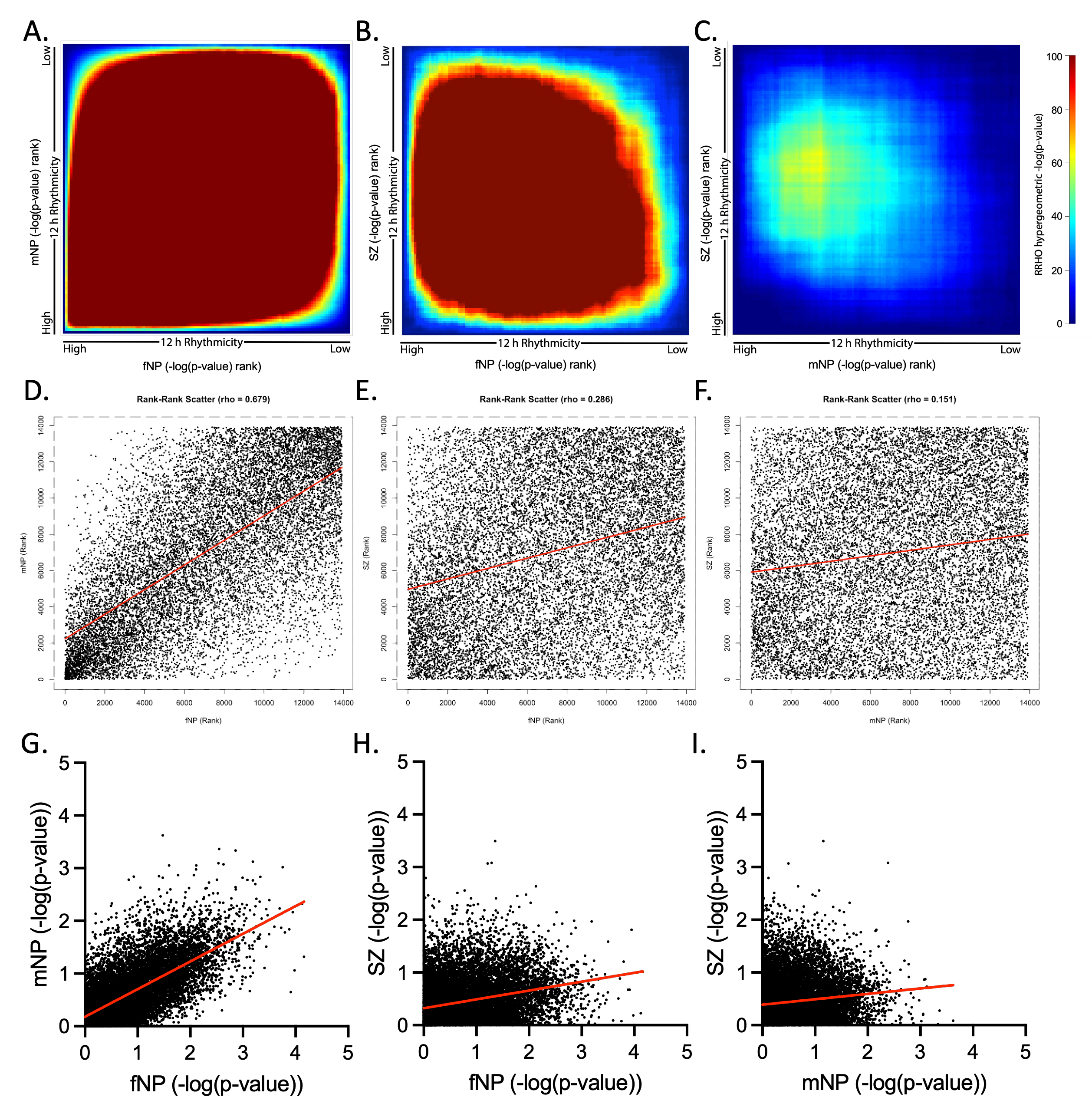

Supplement: S6 Fig — RRHO plots comparing the (A) full and match NP cohorts, the (B) fNP and SZ cohorts, and the (C) mNP and SZ cohorts. (D-F) Corresponding Rank-Rank scatterplots for each cohort comparison are shown. These show each genes p-value rank within each cohort compared to the other cohorts. (G-I) Scatterplots comparing the original -log(p-values) for each gene between cohorts. These plots were created with information that can be found in S1 File. fNP, full NP; mNP, match NP; NP, nonpsychiatric; RRHO, rank-rank hypergeometric overlap; SZ, schizophrenia. (TIFF) [file pbio.3001688.s006.tiff]

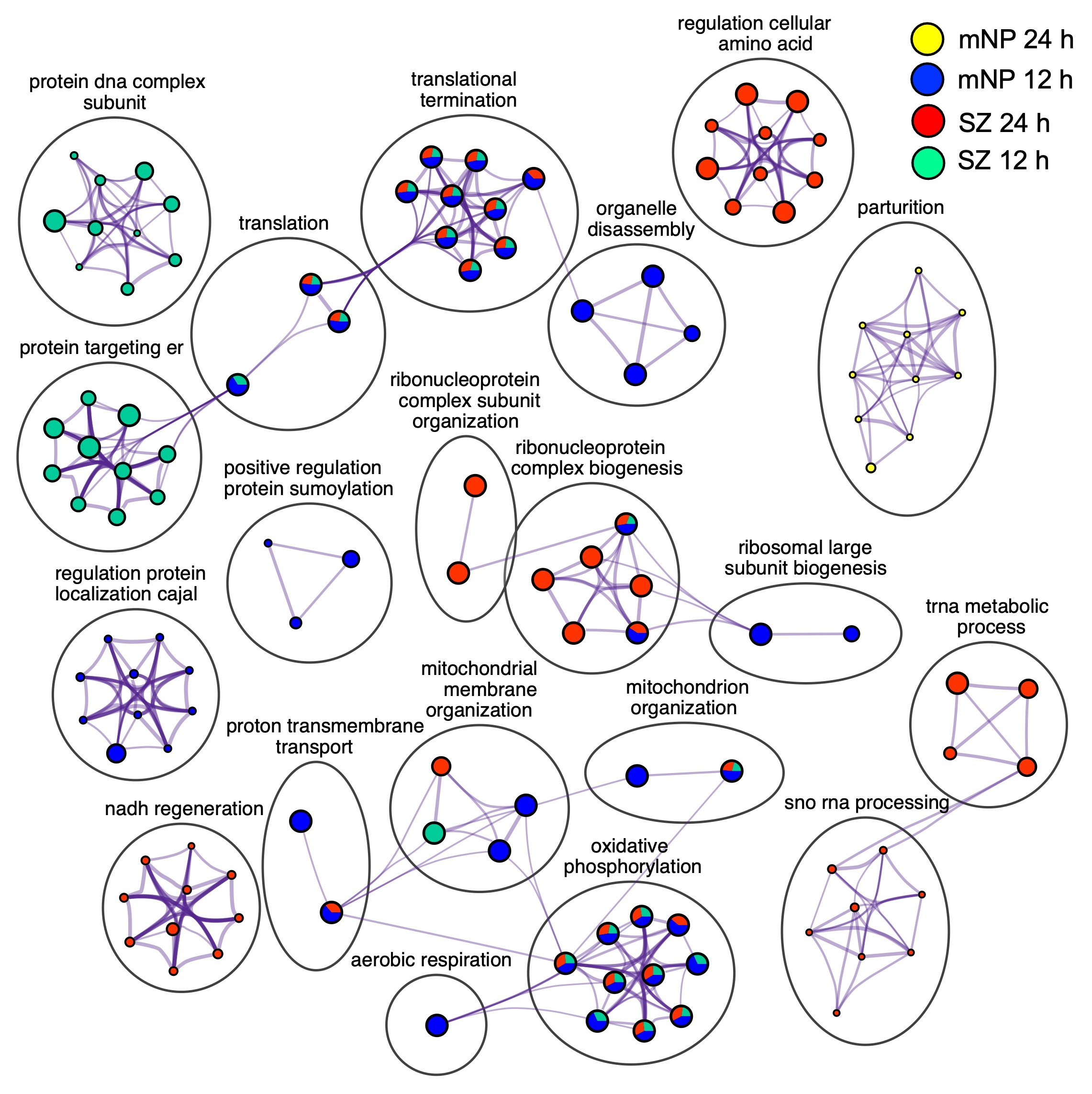

Supplement: S7 Fig — Cytoscape depiction of a Metascape analysis of transcripts with 12 and 24 h rhythms in both the mNP and SZ cohorts. Metascape results can be found in S11 File. mNP, match NP; SZ, schizophrenia. (TIFF) [file pbio.3001688.s007.tiff]

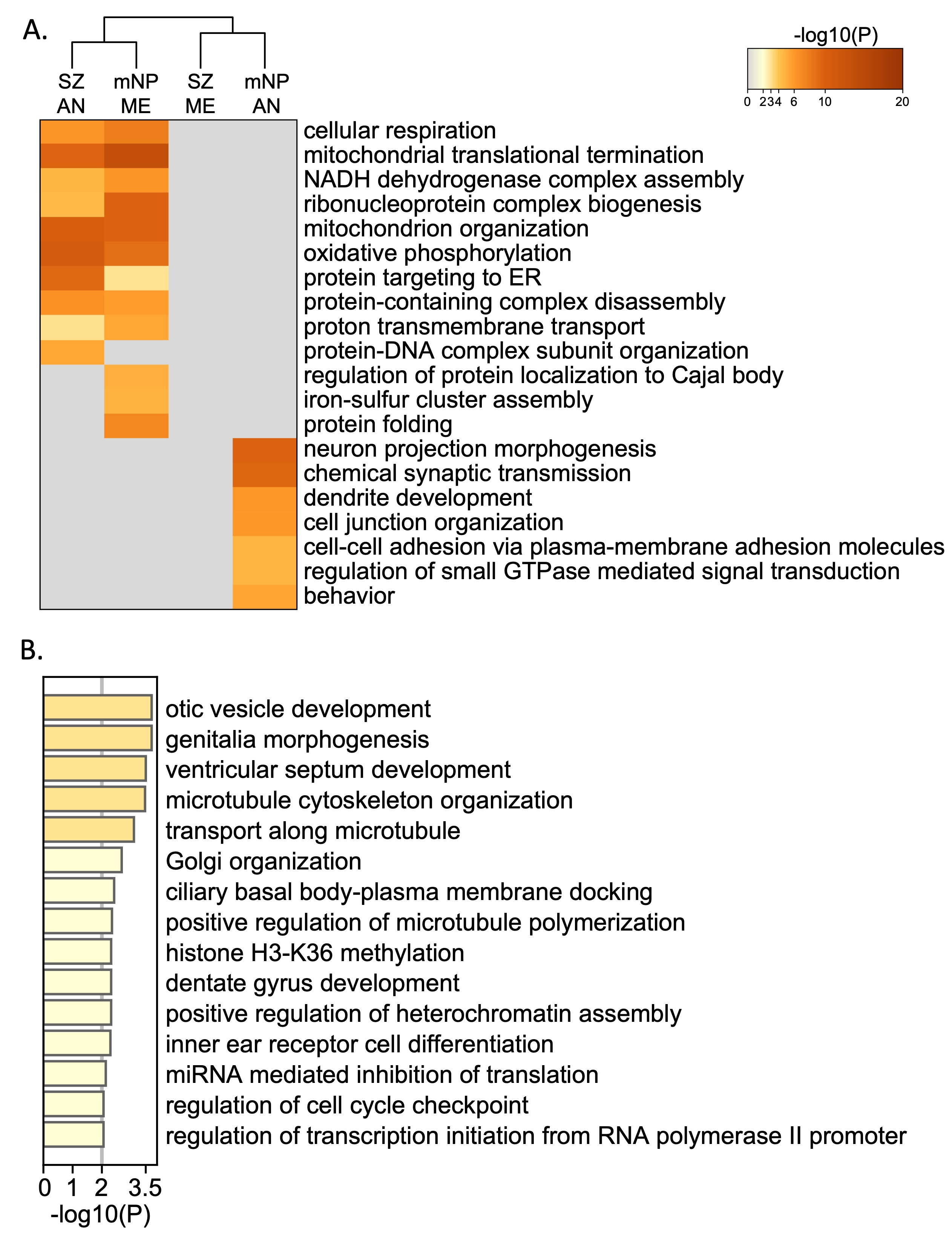

Supplement: S8 Fig — (A) Heatmap of Metascape analysis of transcripts with 12 h rhythms that peak in expression either in the ME or AN. (B) Biological processes enriched in the SZ ME group analyzed separately due to much lower strength of enrichment than the other 3 groups. Metascape results can be found in S11 File. AN, afternoon/night; ME, morning/evening; SZ, schizophrenia. (TIFF) [file pbio.3001688.s008.tiff]

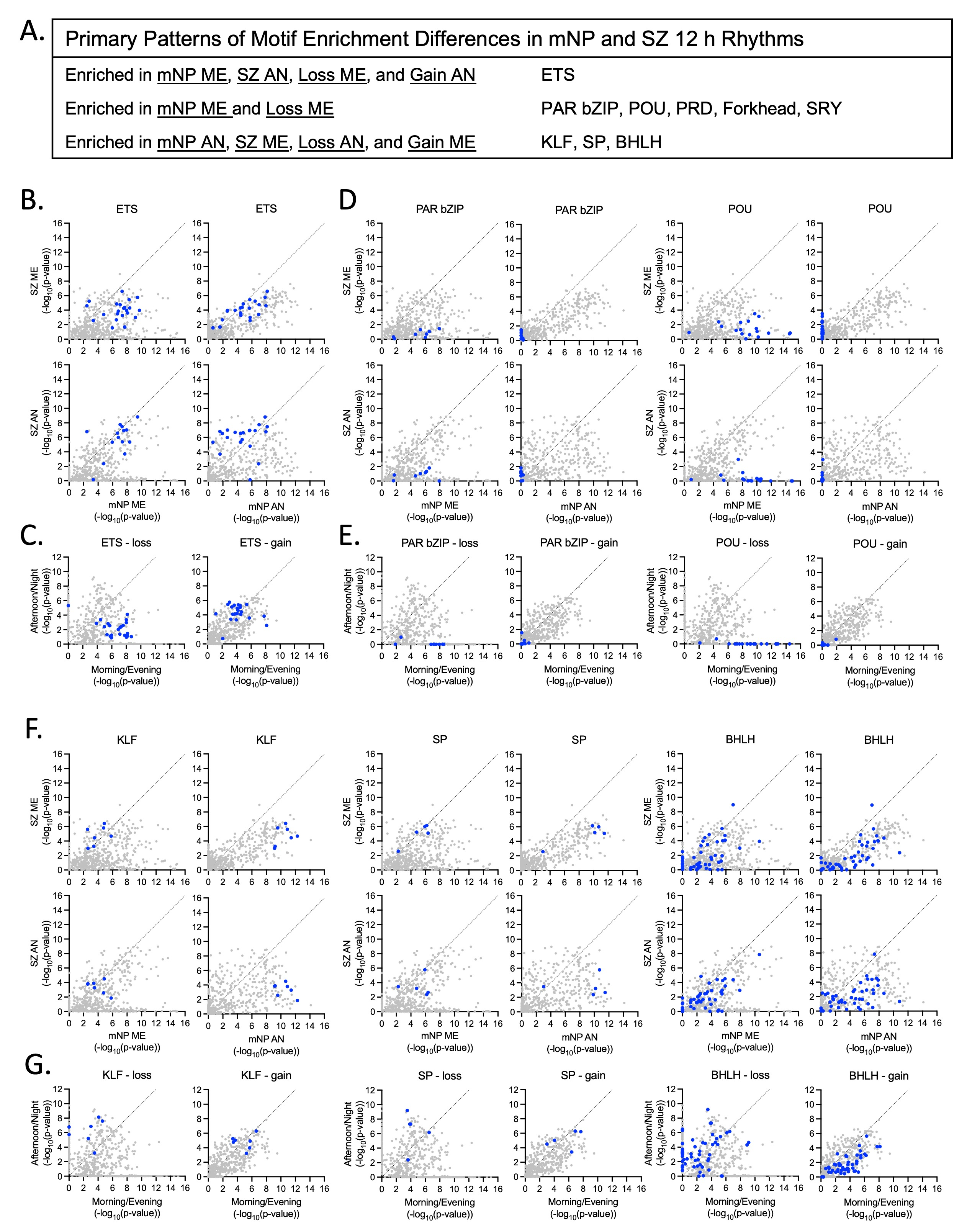

Supplement: S9 Fig — (A) Summary of patterns in protein families associated that emerge after motif enrichment analysis. Families include ETS domain family (ETS), PAR bZIP family, POU class homeoboxes, PRD class homeoboxes, Forkhead boxes, SRY boxes, KLFs, SP domain family (SP), BHLH domain family (BHLH). (B, D, F) Comparison of motif enrichment scores (-log10(pvalue) between mNP and SZ for transcripts that peak either in the ME or AN. (C, E, G) Comparison of motif enrichment scores between ME and AN groups for transcripts that either significantly lose 12 h rhythms in SZ or significantly gain 12 h rhythms in SZ. (B-C) ETS domain family, which shows altered timing in SZ (mNP ME to SZ AN). (D-E) Examples of protein families have enrichment in mNP ME, but no enrichment in any SZ groups. (F-G) Examples of protein families with altered timing in SZ (mNP AN to SZ ME). Plots in this figure were created from data that can be found in S8 File. AN, afternoon/night; BHLH, Basic Helix–Loop–Helix; bZIP, basic leucine zipper; KLF, Kruppel-like factor; ME, morning/evening; mNP, match NP; SRY, Sex determining region Y; SZ, schizophrenia. (TIFF) [file pbio.3001688.s009.tiff]
