# Supplementary material for: Twelve-hour rhythms in transcript expression within the human dorsolateral prefrontal cortex are altered in schizophrenia
Source: PLoS Biol. 2023 Jan 24;21(1):e3001688. doi: 10.1371/journal.pbio.3001688 (PMC9873190; doi:10.1371/journal.pbio.3001688)
Supplement: S1 Table — (PDF) [file pbio.3001688.s010.pdf]

	Full NP (n = 104)	Match NP (n =46)	SZ (n = 46)
**Sex (Male/Female)**	81/23	33/13	32/14
**Race (White/Black)**	86/17	35/11	34/12
**Age (y, mean ± SD)**	48.4 ± 12.3	49.1 ± 13.2	50.1 ± 11.5
**PMI (h, mean ± SD)**	17.9 ± 6.0	17.3 ± 6.2	17.0 ± 8.3
**Brain pH (mean ± SD)**	6.7 ± 0.2	6.6 ± 0.2	6.5 ± 0.3
**TOD (mean ± SD)**	8.6 ± 5.7	7.0 ± 6.4	7.8 ± 5.2
**Site (Pitt/MSSM)**	61/43	22/24	22/24

Table S1. Description of CommonMind Consortium cohorts
Abbreviations: y = years, h = hours; PMI = postmortem interval; SD = standard deviation; TOD = time of death;
