# Supplementary material for: Twelve-hour rhythms in transcript expression within the human dorsolateral prefrontal cortex are altered in schizophrenia
Source: PLoS Biol. 2023 Jan 24;21(1):e3001688. doi: 10.1371/journal.pbio.3001688 (PMC9873190; doi:10.1371/journal.pbio.3001688)
Supplement: S2 Table — (PDF) [file pbio.3001688.s011.pdf]

		p < 0.05	p < 0.01	p < 0.001
**Full NP (n = 104)**	**24 h**	2333	607	83
	**12 h**	2891	819	58
**Match NP (n = 46)**	**24 h**	647	81	7
	**12 h**	1399	186	9
**SZ (n = 46)**	**24 h**	801	91	7
	**12 h**	576	51	3

Table S2. Number of transcripts with significant 12 or 24 h rhythms identified by a sinusoidal nonlinear regression
